# PGE2 mediates EGFR internalization and nuclear translocation *via* caveolin endocytosis promoting its transcriptional activity and proliferation in human NSCLC cells

**DOI:** 10.18632/oncotarget.24499

**Published:** 2018-02-15

**Authors:** Lorenzo Bazzani, Sandra Donnini, Antonio Giachetti, Gerhard Christofori, Marina Ziche

**Affiliations:** ^1^ Department of Life Sciences, University of Siena, Siena, Italy; ^2^ Department of Biomedicine, University of Basel, Basel, Switzerland

**Keywords:** nuclear EGFR, PGE_2_, clathrin and caveolin endocytosis, gene transcription, cell proliferation

## Abstract

Prostaglandin E_2_ (PGE_2_) contributes to tumor progression by promoting cancer cell growth, invasion and by creating a favorable pro-tumor microenvironment. PGE_2_ has been reported to transactivate and internalize into the nucleus receptor tyrosine kinases such as Epidermal growth factor receptor (EGFR), thereby supporting tumor progression. Here we demonstrate that in non-small cell lung carcinoma (NSCLC) cells, PGE_2_ induces EGFR nuclear translocation via different dynamin-dependent endocytic pathways, promotes the formation of an EGFR-STAT3 complex, affects nuclear EGFR target gene expression and mediates tumor cell proliferation. Indeed, we find that PGE_2_ induces EGFR internalization and consequent nuclear import through Clathrin- and Caveolin-mediated endocytosis and through the interaction of EGFR with Importin β1. Within the nucleus, EGFR forms a complex with STAT3, an event blocked by ablation of Clathrin Heavy Chain or Caveolin-1. The combination of EGF and PGE_2_ prolongs nuclear EGFR transcriptional activity manifested by the upregulation of *CCND1*, *PTGS2*, *MYC* and *NOS2* mRNA levels and potentiates nuclear EGFR-induced NSCLC cell proliferation. Additionally, NSCLC patients with high expression of a nuclear EGFR gene signature display shorter survival times than those with low expression, thus showing a putative correlation between nuclear EGFR and poor prognosis in NSCLC. Together, our findings indicate a complex mechanism underlying PGE_2_-induced EGF/EGFR signaling and transcriptional control, which plays a key role in cancer progression.

## INTRODUCTION

Prostaglandin E_2_ (PGE_2_) promotes tumor growth by inducing an inflammatory microenvironment, in autocrine or paracrine fashion, through the activation of 4 receptor subtypes: EP1, EP2, EP3, EP4 [1–3]. Besides the plethora of biological processes evoked by prostanoids through the binding to their receptors, PGE_2_ reportedly interacts with various receptor tyrosine kinase (RTK) in a process termed transactivation [4], exemplified by EGFR activation in several cancer cell types [5–10]. Upon binding with PGE_2_, EP receptors trigger different downstream effectors including PKA, PKC, SRC and PI3K to mediate EGFR activation [11].

We have recently reported that PGE_2_ induces EGFR internalization and nuclear translocation supporting tumor progression in non-small cell lung cancer (NSCLC) cells [12]. Indeed, we have shown that PGE_2_-induced EGFR transactivation promotes its nuclear import and the subsequent SRC/ADAMs-mediated autocrine and/or paracrine release of soluble cell-surface EGF like ligands, an event that culminates in EGFR-mediated transcriptional activities and enhanced tumor cell proliferation [12].

Numerous reports have shown that EGFR recruits proteins and transduces signaling pathways also inside the cell [13, 14]. Upon ligand binding, EGFR undergoes either Clathrin-mediated endocytosis (CME) or Clathrin-independent endocytosis (CIE), including lipid-raft dependent routes, such as Caveolin-mediated endocytosis and macropinocytosis [15]. EGFR is mainly internalized via Clathrin- mediated endocytosis [16], yet saturation of Clathrin or stimulation with different ligands has been shown to induce alternative routes of internalization, including Caveolin-mediated endocytosis and macropinocytosis [17–19].

In light of the above mentioned PGE_2_ -induced EGFR nuclear translocation, we have tested which internalization routes might be involved in EGFR nuclear shuttling in NSCLC cells and whether PGE_2_ could sustain nuclear EGFR transcriptional activity and tumor progression. Here, we describe the internalization mechanisms by which PGE_2_ regulates EGFR nuclear translocation and affects tumor gene expression and cancer cell proliferation.

## RESULTS

### Inhibition of the large GTPase dynamin blocks PGE_2_-induced EGFR nuclear import

**Figure 1:**
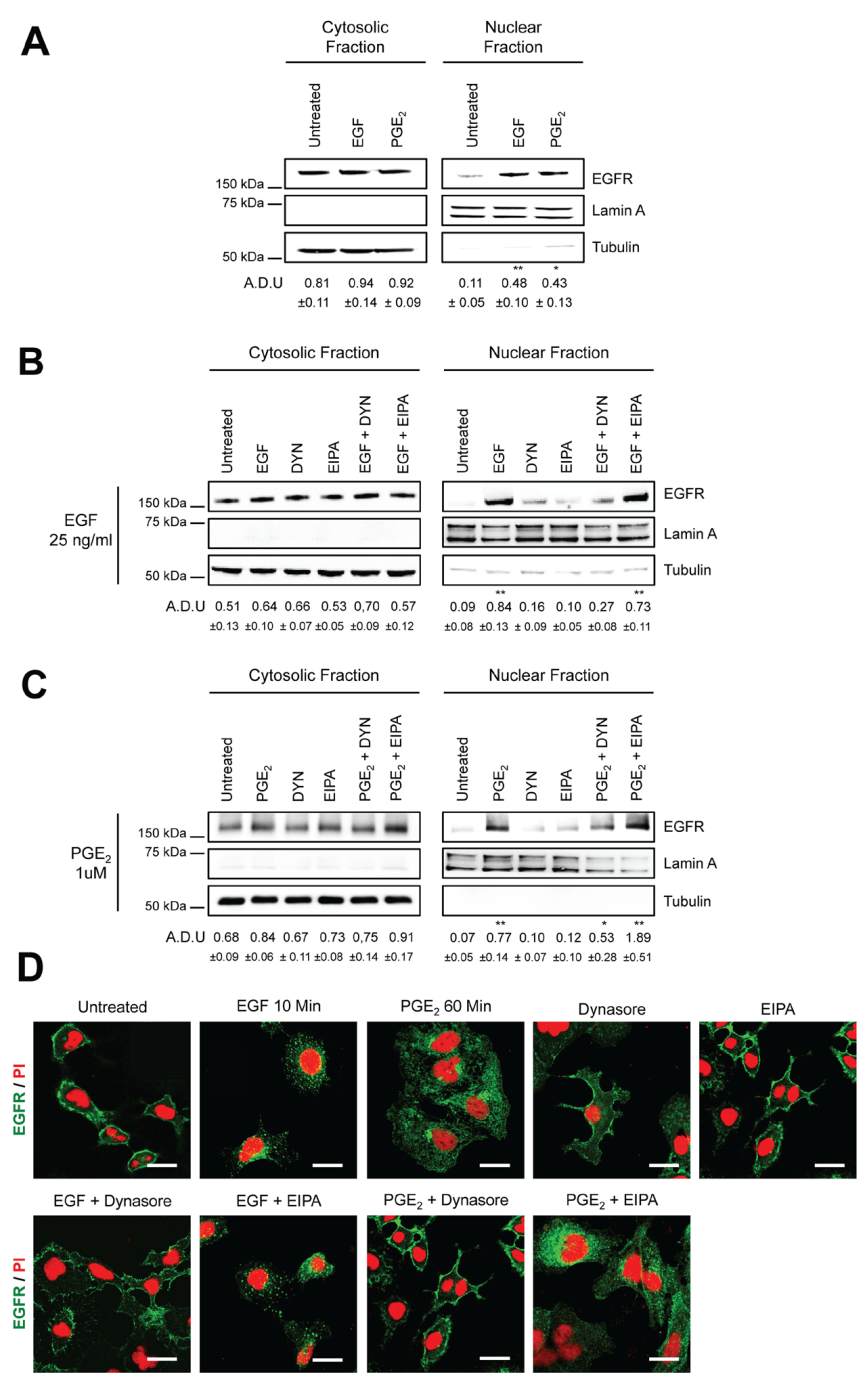
Dynamin inhibition blocks EGF- and PGE2-induced EGFR nuclear translocation Immunoblotting analysis of EGFR expression in cytosolic and nuclear fraction in overnight starved A549 **(A–C)**. Cells were exposed to 10 min to 25ng/ml EGF or to 60 min 1μM PGE_2_ (A). A549 cells were starved overnight and then treated for 30 min with dynasore 80μM (DYN) or 100μM 5-(N-Ethyl-N- isopropyl)amiloride (EIPA) before challenge with 25ng/ml EGF or 1μM PGE_2_ for 10 and 60 min respectively (B, C). Tubulin and Lamin A were used as loading control for cytosolic and nuclear fraction. Immunoblotting quantification was expressed in A.D.U. (arbitrary density unit) and as mean ± SD. ^*^p < 0.05, ^**^p < 0.01vs Ctrl. EGFR in the cytoplasmic and nuclear fractions was normalized to Tubulin or Lamin A respectively. **(D)** Confocal analysis of EGFR localization in A549 treated as described above and then fixed with paraformaldehyde and stained with anti-EGFR (*green*) and Propidium Iodide (*red*). Confocal images were captured in the middle section of the nuclei with Zeiss LSM700 microscope using 63x objective, scale bars 20 μm. The experiments were performed three times.

EGFR is internalized either via Clathrin-mediated endocytosis or via Caveolin-mediated endocytosis and macropinocytosis [16–18, 20]. In order to discriminate between the EGFR endocytic modalities induced by PGE_2_, we used pharmacological inhibitors of these pathways employing A549 and GLC82 NSCLC cells. Stimulation with either EGF (25 ng/ml) or PGE_2_ (1μM) induced EGFR nuclear translocation with a peak at 10 and 60 min, respectively (Figure 1A and Supplementary Figure 1A). Clathrin- and Caveolin-mediated endocytosis are both dependent on the activity of the large guanosine 5′-triphosphatase (GTPase) dynamin [21], whereas macropinocytosis is susceptible to Na+/H+ exchange inhibitors, such as amiloride [22]. Thus, A549 cells were pre-treated with a specific dynamin inhibitor, Dynasore (DYN), or with a Na+/H+ exchange inhibitor, 5-(N-Ethyl-N-isopropyl) amiloride (EIPA) before challenge with EGF (Figure 1B) or PGE_2_ (Figure 1C). In EGF or PGE_2_-treated cells, DYN markedly reduced EGFR nuclear accumulation, whereas no effect was observed with EIPA (Figure 1B and 1C). Similar results were obtained with GLC82 cells (Supplementary Figure 1B). Immunofluorescence staining followed by confocal microscopy analysis showed that in control conditions and in NSCLC cells treated with DYN or EIPA alone, EGFR was confined to the cell membrane (Figure 1D). DYN pretreatment of NSCLC cells blocked the nuclear import of EGFR induced by either EGF (10 min) or PGE_2_ (60 min), whereas EIPA treatment failed to affect the EGFR internalization and nuclear translocation process (Figure 1D). The results indicate that dynamin-dependent Clathrin- and Caveolin-mediated endocytosis might be involved in EGF and PGE_2_-induced EGFR nuclear translocation.

### PGE_2_ promotes EGFR internalization and nuclear translocation via Clathrin- and Caveolin-mediated endocytosis

**Figure 2:**
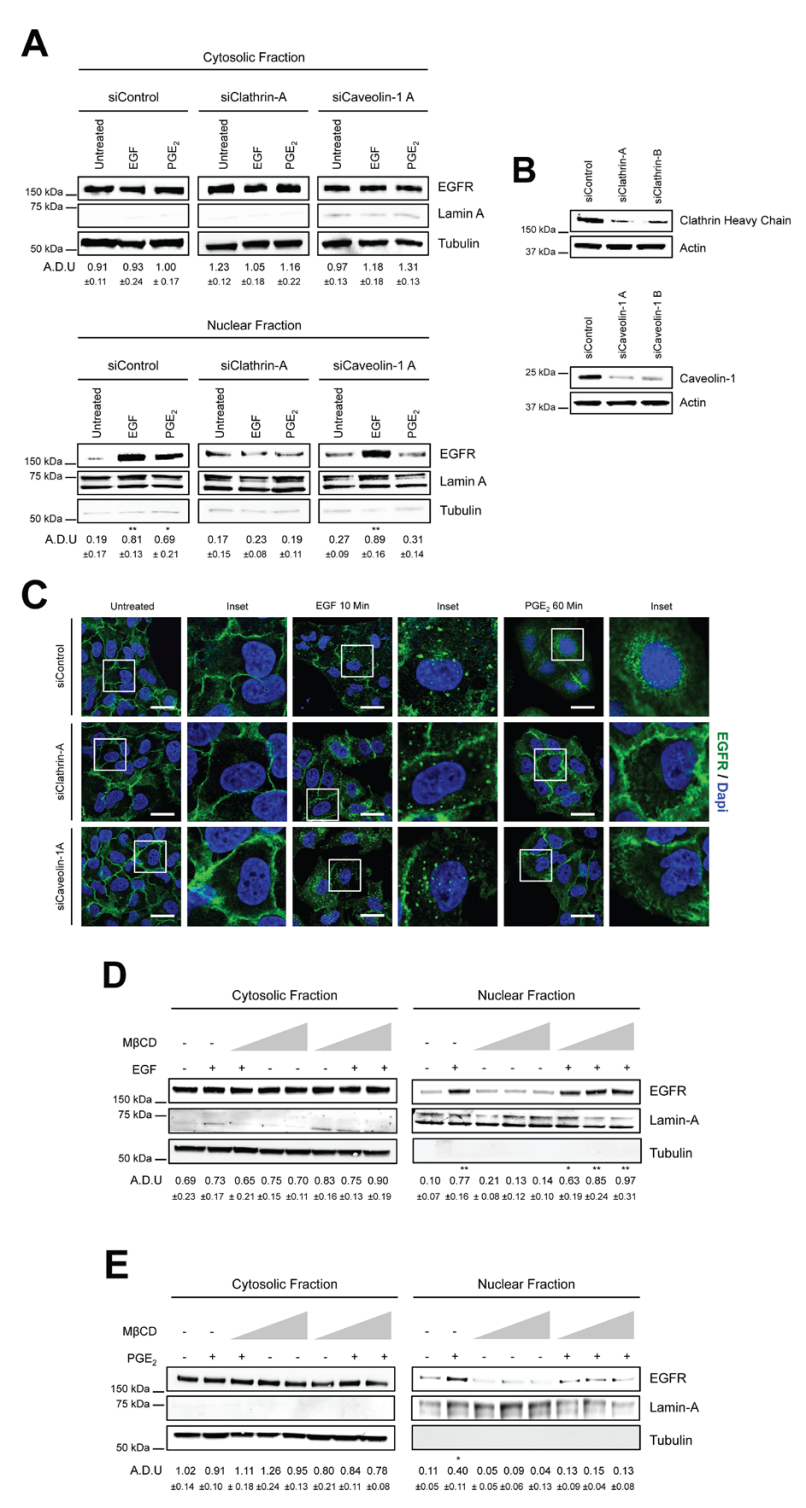
PGE_2_ promotes EGFR internalization via Clathrin- and Caveolin-mediated endocytosis **(A)** A549 cells were transfected with siRNA control or siRNAs against Clathrin Heavy Chain or Caveolin-1 for 24 h. Cells were then serum starved overnight and then exposed to 25ng/ml EGF for 10 min or to 1μM PGE_2_ for 60 min. EGFR level in cytoplasmic and nuclear fraction was assessed using immunoblot with indicated antibodies. Tubulin and Lamin A were used as loading control for cytosolic and nuclear fraction. **(B)** Knockdown efficiency was verified by immunoblotting with Clathrin Heavy Chain or Caveolin-1 antibodies, actin was used as loading control. Data are shown only for siClathrin-A and siCaveolin-1A, similar data were obtained with siClathrin-B and siCaveolin-1B. **(C)** 48 h post transfection, cells were treated with EGF or PGE_2_ as indicated in the panels, fixed and stained for EGFR (*green*) and DAPI (*blue*). Pictures were acquired in the middle section of nuclei at 63x magnification. Scale bars, 20μm. Panel shows representative picture for each experimental condition. Boxed areas are shown in detail in the inset. **(D-E)** A549 cells were starved overnight and then treated with Metil β-cyclodextrin (MβCD) 1, 5 and 10 mM for 30 min before challenge with 25ng/ml EGF (D) or 1μM PGE_2_ (E) for 10 and 60 min respectively. Immunoblotting analysis on cytoplasmic and nuclear fractions was then performed with indicated antibodies. Immunoblotting quantification was expressed in A.D.U. (arbitrary density unit) and as mean ± SD. ^*^p < 0.05, ^**^p < 0.01 vs Ctrl. EGFR in the cytoplasmic and nuclear fractions was normalized to Tubulin or Lamin A respectively. The experiments were performed three times.

To further dissect the endocytic mechanism, we used a genetic approach to inhibit Clathrin and Caveolin-mediated endocytosis by performing knockdown of both Clathrin Heavy Chain, the major component of Clathrin-coated vesicles [23] and knockdown of Caveolin-1, the primary constituent of Caveolae [24]. A549 and GLC82 cells were transiently transfected with siRNA against Clathrin Heavy Chain (siClathrin) or against Caveolin-1 (siCaveolin-1) or with a non-targeting siRNA (siControl) for 48 hours and then treated with EGF (10 min) or PGE_2_ (60 min). Clathrin knockdown abrogated EGFR nuclear translocation induced by both EGF and PGE_2_, while Caveolin-1 ablation suppressed only PGE_2_-induced EGFR nuclear translocation (Figure 2A). The efficient knockdown of Clathrin Heavy chain and Caveolin-1 expression was confirmed by immunoblotting (Figure 2B). A similar phenotype was observed in GLC82 cells. Clathrin Heavy chain knockdown hindered both EGF and PGE_2_-mediated EGFR nuclear translocation. Conversely, knockdown of Caveolin-1 impaired only PGE_2_-mediated EGFR nuclear accumulation (Supplementary Figure 2A and 2B). Immunofluorescence confocal microscopy analysis confirmed the strong reduction of EGFR internalization and nuclear translocation in Clathrin Heavy chain knockdown setting (Figure 2C middle panels) and the dependency on Caveolin-mediated endocytosis of PGE_2_-induced EGFR nuclear translocation (Figure 2C lower panels). Knockdown of both Clathrin Heavy chain and Caveolin-1, impaired PGE_2_ activity to a similar extent as si-Clathrin Heavy chain or si-Caveolin-1 alone, suggesting that the prostanoid appears to promote nuclear EGFR translocation with two independent signaling pathways (Supplementary Figure 3A). The efficient knockdown of Clathrin Heavy chain and Caveolin-1 expression was confirmed by immunoblotting (Supplementary Figure 3B).

To validate the results on Caveolin-mediated endocytosis, we employed Methyl-l β-cyclodextrin (MβCD), a cyclic oligomer of glucopyranoside that inhibits cholesterol-dependent Caveolae-mediated endocytosis, by reversibly extracting the steroid out of lipid rafts [25]. In A549 cells, incubated with increasing concentration of MβCD (1, 5, 10 mM) before EGF or PGE_2_ treatment, we found no effect on EGF-mediated EGFR nuclear translocation (Figure 2D), whereas a reduced EGFR nuclear accumulation was observed after PGE_2_ stimulation, demonstrating that Caveolin-mediated endocytosis plays a role in PGE_2_-induced EGFR nuclear translocation (Figure 2E). Taken together, these findings underline the prominent role of Clathrin-mediated endocytosis in EGFR internalization, yet the data also demonstrates that Caveolin-mediated endocytosis is an alternative endocytic route for PGE_2_-induced nuclear translocation of EGFR.

### Importin β1 is essential for PGE_2_-mediated EGFR nuclear translocation

**Figure 3:**
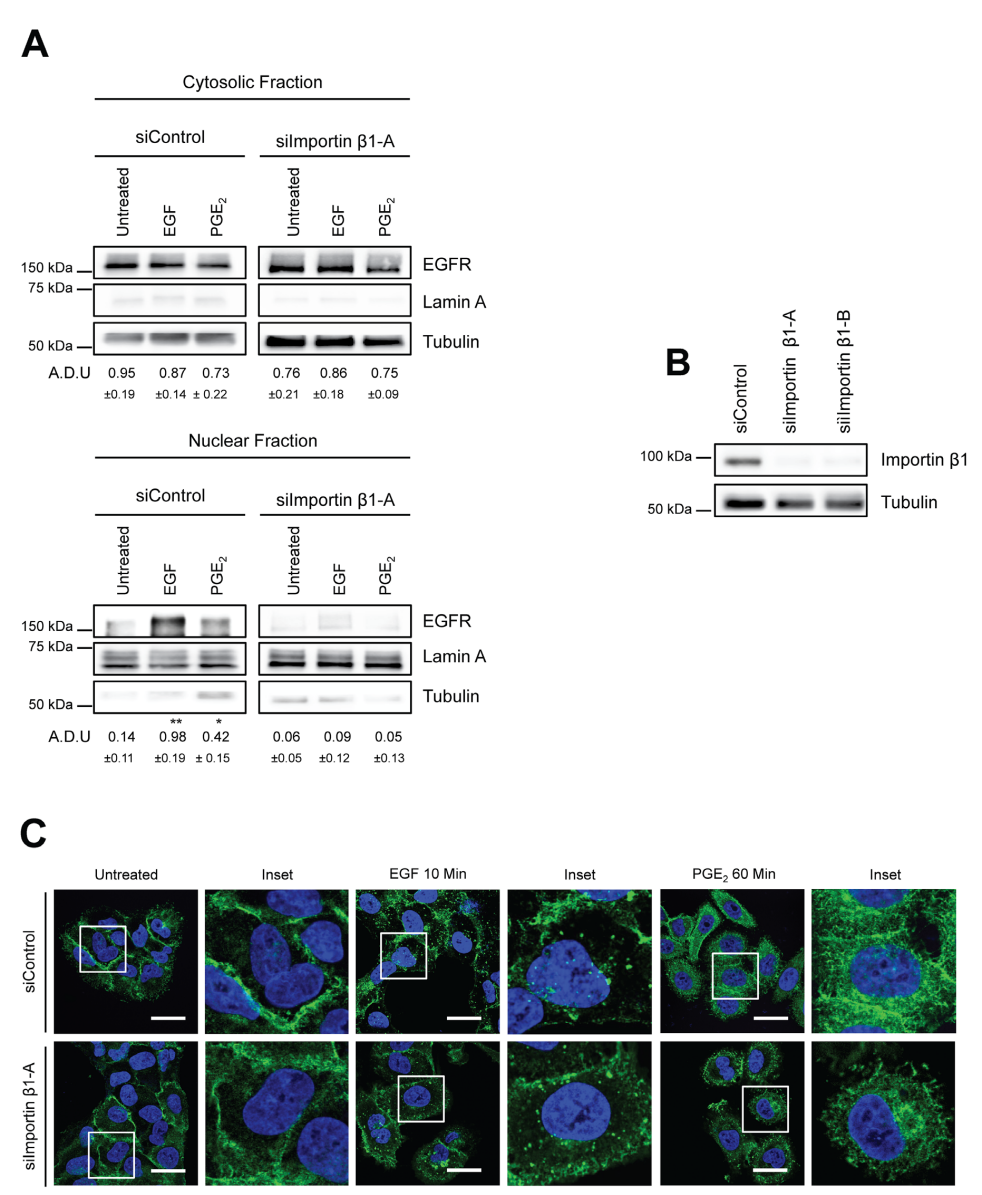
Importin β1 is required for EGFR nuclear translocation **(A)** A549 cells were transfected with siRNA control or siRNA against Importin β1 for 48h. Next, cells were serum starved overnight and exposed to 25ng/ml EGF for 10 min or to 1μM PGE_2_ for 60 min. EGFR level in cytoplasmic and nuclear fraction was assessed using immunoblot with indicated antibodies. **(B)** Knockdown efficiency was verified via western blot with Importin β1 antibody, Tubulin was used as loading control. Similar data were obtained with siImportin β1-B. Immunoblotting quantification was expressed in A.D.U. (arbitrary density unit) and as mean ± SD. ^*^p < 0.05, ^**^p < 0.01 vs Ctrl. EGFR in the cytoplasmic and nuclear fractions was normalized to Tubulin or Lamin A respectively. **(C)** A549 cells were transfected as indicated above. After that cells were fixed and stained for EGFR (*green*) and DAPI (*blue*). Confocal images were captured in the middle section of the nuclei with Leica SP5 confocal using 63x objective, scale bars 20 μm. Panel shows representative picture for each experimental condition. Boxed areas are shown in detail in the inset. The experiments were performed three times.

Transport of proteins into the nucleus through the nuclear pore complex involves Importin α/β, which bind to nuclear localization signals in cargo substrates to promote nuclear entry [26]. Indeed, a putative tripartite NLS is present within the juxtamembrane domain of EGFR and is required for its nuclear import via association with Importin β1 [27, 28]. To assess whether Importin β1 was essential for PGE_2_-mediated EGFR nuclear translocation, as demonstrated for EGF, we performed a knockdown of Importin β1 by transfecting A549 cells with siRNA against Importin β1 or with a non-targeting siRNA (siControl). Exposure to EGF or PGE_2_ of Importin β1-silenced cells dramatically reduced EGFR nuclear translocation upon both EGF and PGE_2_ treatments (Figure 3A). Downregulation of Importin β1 was assessed by immunoblotting analysis (Figure 3B). The role of Importin β1 was further validated by immunofluorescence confocal microscopy analysis. In Importin β1-ablated cells, EGFR was still mobilized from cell membrane upon both EGF and PGE_2_ treatment, however, nuclear localization was abrogated (Figure 3C). These results demonstrate that, as for EGF, PGE_2_-mediated EGFR nuclear translocation requires Importin β1.

### PGE_2_ induces the formation of an EGFR-STAT3 complex in the nucleus

**Figure 4:**
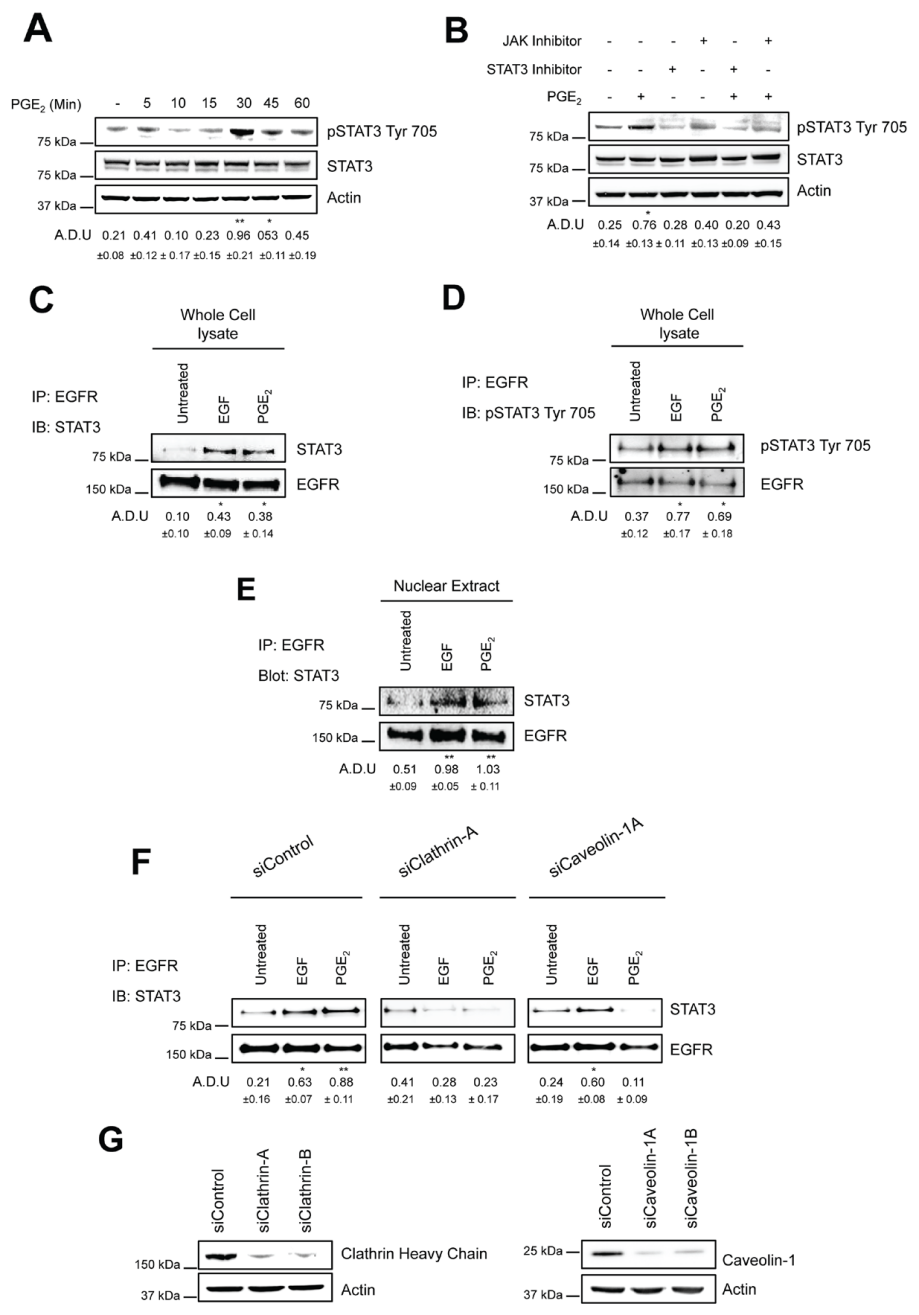
PGE_2_ induces the formation of EGFR-STAT3 complex into the nucleus **(A)** Immunoblotting analysis of STAT3 phosphorylation on Tyr 705 in overnight starved A549 exposed to 1 μM PGE_2_ for 5–60 min. **(B)** Immunoblotting analysis of STAT3 phosphorylation on Tyr 705 in A549 exposed for 30 min to 1 μM PGE_2_, with or without pre-incubation with JAK inhibitor, Ruxolitinib (10 μM), or STAT3 inhibitor, STAT3 inhibitor VII, for 30 min. Actin was used as loading control. **(C–E)** A549 cells were overnight starved and then exposed to 25ng/ml EGF or 1μM PGE_2_ for 10 and 60 min respectively. Whole cell lysate (C-D) and nuclear extract (E) were subjected to immunoprecipitation with anti-EGFR antibody and analyzed by immunoblotting with anti-STAT3 or anti-phosphoSTAT3 Try 705 antibodies. **(F)** A549 cells were transfected with siRNA Control or siRNA against Clathrin Heavy Chain or against Caveolin-1 for 24 h. Cells were then serum starved overnight and treated with 25ng/ml EGF for 10 min or 1μM PGE_2_ for 60 min. Whole cell lysates were subjected to immunoprecipitation with anti-EGFR antibody and analyzed by immunoblotting with anti-STAT3 antibody. **(G)** Knockdown efficiency was verified via western blot with Clathrin heavy chain and Caveolin-1 antibodies, actin was used as loading control. Data are shown only for siClathrin-A and siCaveolin-1A, similar data were obtained with siClathrin-B and siCaveolin-1B. Immunoblotting quantification was expressed in A.D.U. (arbitrary density unit) and as mean ± SD. ^*^p < 0.05, ^**^p < 0.01 vs Ctrl. In panel A and B, pSTAT3 Tyr 705 was normalized to STAT3. In panels C-F, STAT3 was normalized to EGFR. The experiments were performed three times.

As EGFR lacks a DNA binding domain, nuclear EGFR exerts its transcriptional functions via the interaction with various transcription factors [29]. Among the transcriptional target genes, nuclear EGFR is recruited to the ATRS motif of the cyclin D1 (*CCND1*), inducible nitric oxide synthase (*NOS2*), c-Myc (*MYC*) and COX-2 (*PTGS2*) promoters through its interaction with several transcription factors, including STAT3 [30–34]. We previously observed that *CCND1*, *PTGS2*, *MYC* and *NOS2* were highly modulated by PGE_2_-induced nuclear EGFR [12]. Thus, we first investigated whether PGE_2_ promoted STAT3 phosphorylation and then the role of JAK in mediating this signaling pathway. In A549 cells exposed to PGE_2_ from 5 to 60 min, the prostanoid induced STAT3 activation in a time-dependent manner with a peak at 30 min (Figure 4A). Next, 30 minutes pre-treatment of NSCLC cells with a JAK inhibitor, Ruxolitinib at 10μM, as well as with a STAT3 inhibitor, STAT3 inhibitor VII at 10μM, significantly reduced the PGE_2_ effect (Figure 4B).

Notably, PGE_2_ promoted EGFR-STAT3 protein–protein interaction as determined by co-immunoprecipitation experiments. A549 cells were exposed to EGF (10 min) or PGE_2_ (60 min), and EGFR was immunoprecipitated and the potential binding of STAT3 to EGFR was analyzed by immunoblotting. Upon EGF and PGE_2_ treatment, STAT3, either in its non-phosphorylated or phosphorylated condition, efficiently co-immunoprecipitated with EGFR (Figure 4C, 4D). In order to show whether EGFR and STAT3 form a complex inside the nucleus, we performed cell fractionation followed by immunoprecipitation. We observed that EGFR was bound to STAT3 in the nucleus suggesting a possible mechanism for the transcriptional activation of the *PTGS2*, *MYC* and *NOS2* genes (Figure 4E). To assess the contribution of the two above-described internalization routes on EGFR transcriptional activity, we transiently transfected A549 and GLC82 cells with siRNAs against Clathrin Heavy Chain (siClathrin) or against Caveolin-1 (siCaveolin-1) or with a non-targeting siRNA (siControl) for 48 hours before treatment with EGF (10 min) or PGE_2_ (60 min) (Figure 4F and Supplementary Figure 4A). In Clathrin-ablated A549 cells exposed to EGF or PGE_2_, the formation of an EGFR-STAT3 complex was significantly reduced, confirming the central role of Clathrin-mediated endocytosis in EGFR internalization (Figure 4F and Supplementary Figure 4A middle panels). However, Caveolin-1 silencing affected PGE_2_-induced EGFR-STAT3 association, corroborating the ability of the prostanoid to use an alternative endocytic pathway (Figure 4F and Supplementary Figure 4A right panels). The knockdown efficiency of Clathrin Heavy chain and Caveolin-1 was confirmed by immunoblotting analysis blot (Figure 4G and Supplementary Figure 4B). The simultaneous knockdown of both Clathrin Heavy chain and Caveolin-1 did not appear to modify PGE_2_ efficacy on EGFR-STAT3 nuclear association, compared to knockdown of Clathrin Heavy chain or Caveolin-1 alone (Supplementary Figure 5A). The knockdown efficiency of Clathrin Heavy chain and Caveolin-1 was confirmed by immunoblotting analysis blot (Supplementary Figure 5B). Taken together, our data demonstrate that PGE_2_ induces the formation of an EGFR-STAT3 nuclear complex and that different endocytic mechanisms contribute to the association of EGFR with STAT3.

### Combination of EGF and PGE2 promotes the transcription of nuclear EGFR target genes up to 8 hours

**Figure 5:**
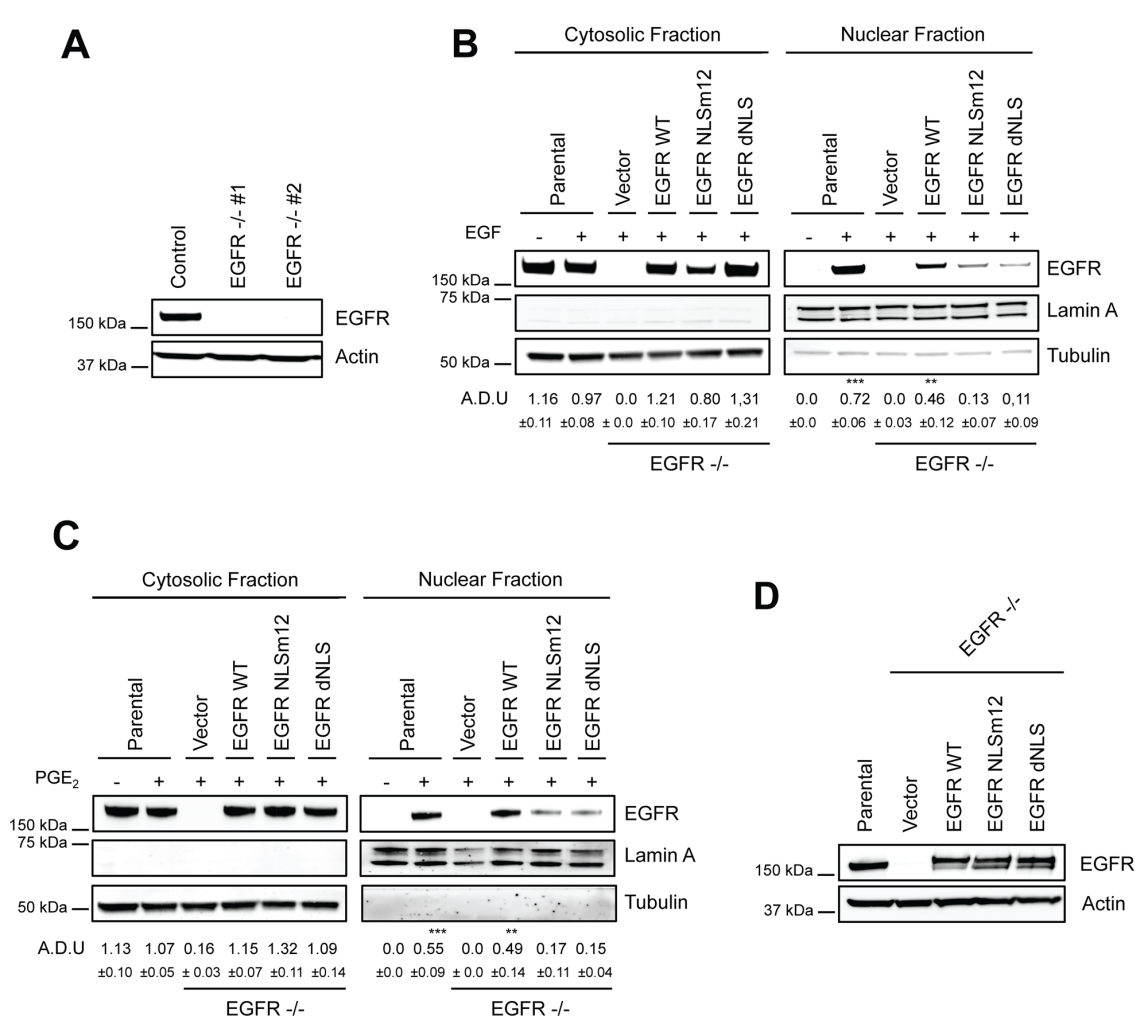
Generation and characterization of a model to study nuclear EGFR functions **(A)** Immunoblotting analysis of EGFR expression in A549 wild type cells and two clones knockout for EGFR, generated by CRISPR/Cas9 (EGFR -/- #1, #2). Actin was used as loading control. **(B–C)** EGFR knockout cells were transiently transfected with Vector or EGFR-WT or EGFR NLS mutant (NLSm12 or dNLS) plasmids for 48 h. Then EGFR nuclear import in response to 25 ng/ml EGF for 10 min (B) or to 1 μM PGE_2_ for 60 min (C) was analyzed by immunoblotting upon cell fractionation. Parental cells were included as a control. Tubulin and Lamin A were used as loading control for cytosolic and nuclear fraction respectively. Immunoblotting quantification was expressed in A.D.U. (arbitrary density unit) and as mean ± SD. ^*^p < 0.05, ^**^p < 0.01, ^***^p < 0.001 vs Ctrl. EGFR in the cytoplasmic and nuclear fractions was normalized to Tubulin or Lamin A respectively. **(D)** Expression of EGFR in EGFR -/- #1 cells, transfected with Vector, EGFR-WT and NLS mutant plasmids for 96 h. Actin was used as loading control. The experiments were performed three times.

We previously reported that EGF and PGE_2_ alone induced the transcription of the nuclear EGFR-target genes *CCND1*, *PTGS2, MYC* and *NOS2,* with a maximal activation at 2 h and 4 h, respectively [12]. To assess the effect of a combination of EGF and PGE_2_ on the expression of these nuclear EGFR transcriptional target genes, A549 EGFR-knockout cells were generated by CRISPR/Cas9 (Figure 5A). Two cell clones, genetically deficient for EGFR expression (EGFR −/− #1 and #2), were transfected with plasmid constructs either encoding for wild type EGFR (WT) or EGFR mutated in its nuclear localization sequence (NLSm12 and dNLS) [32]. In NSCLC cells transfected with NLSm12 or dNLS constructs, EGFR nuclear translocation, promoted by the challenge with EGF or PGE_2_, was markedly reduced compared to cells transfected with WT EGFR or to parental cells (Figure 5B, 5C) [12].

**Figure 6:**
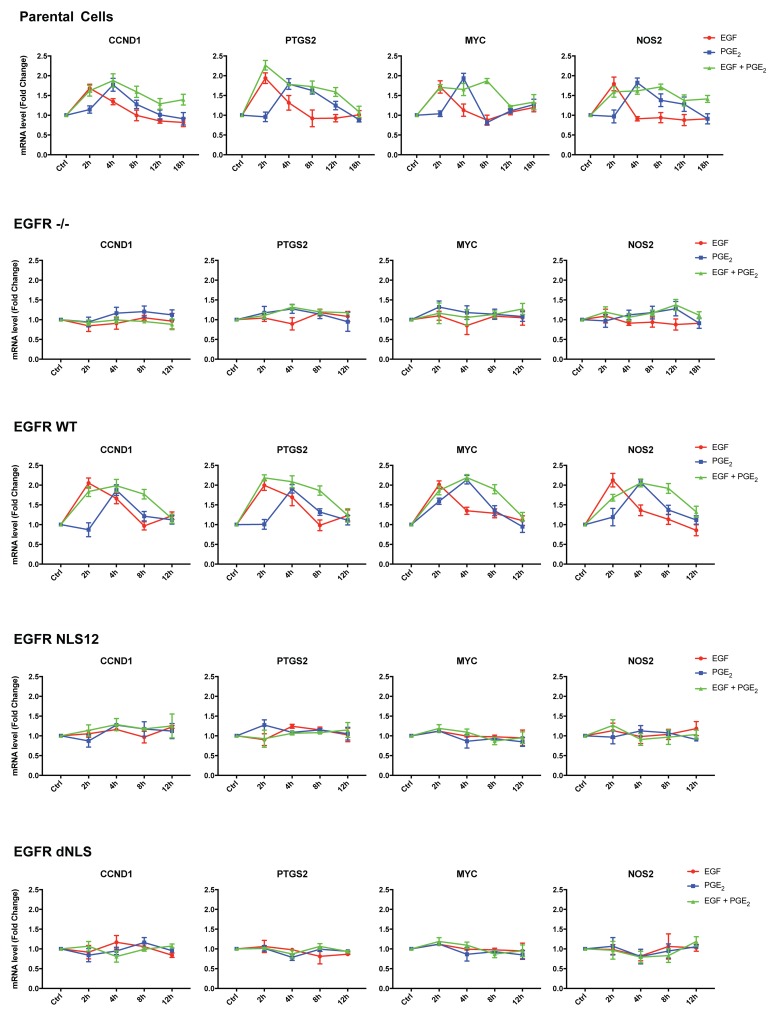
The combination of EGF and PGE_2_ induces the transcription of nuclear EGFR gene signature up to 8 hours Parental and modified A549 cells were starved overnight and then treated with 25 ng/ml EGF or 1 μM PGE_2_ or the combination for 2, 4, 8, 12, or 18 h. RNA was isolated and analyzed by qRT-PCR for a panel of nuclear EGFR target genes. The data are presented as mean of fold change ± SD of three independent experiments, relative to non-treated cells (Control), which were assigned to 1. Statistical analysis is reported in Table 1 and Table 2. The experiments were performed three times.

A549 cells transfected with constructs encoding for WT and mutant EGFR showed similar level of EGFR expression (Figure 5D). Quantitative real-time PCR (qRT-PCR) analysis was performed in parental and modified A549 cells (Figure 6) exposed to EGF or PGE_2_ or a combination in a time course up to 12 or 18 hours. In parental and EGFR -/- A549 cells bearing EGFR WT plasmid, EGF promoted the expression of nuclear EGFR target genes with a peak at 2 h and declined to baseline at 4-8 h, whereas PGE_2_ mimicked the EGF effect on target genes with a peak at 4 h and declined toward the baseline at 8-12 h. The combination of EGF and PGE_2_ recapitulated the outcome of both individual treatments, yet showing a fostered transcription of target genes up to 8 h (Figure 6). Similar results were observed in GLC82 cells (Supplementary Figure 6). The statistical analysis is presented in Tables 1–2 and Supplementary Table 1 for A549 and GLC82 cells, respectively.

**Table 1:** Statistical analysis of nuclear EGFR target genes regulated by EGF and PGE_2_ in parental A549 cells

*CCND1*					
	**2h**	**4h**	**8h**	**12h**	**18h**
EGF vs Ctrl	0.0202 (^*^)	0.0479 (^*^)	0.9818 (ns)	0.1253 (ns)	0.2311 (ns)
PGE_2_ vs Ctrl	0.2561 (ns)	0.0422 (^*^)	0.1211 (ns)	0.9392 (ns)	0.6259 (ns)
EGF+PGE_2_ vs Ctrl	0.0447 (^*^)	0.0353 (^*^)	0.0493 (^*^)	0.2988 (ns)	0.101 (ns)
EGF+PGE_2_ vs EGF	0.731 (ns)	0.1028 (ns)	0.0924 (ns)	0.0983 (ns)	0.0795 (ns)
EGF+PGE_2_ vs PGE_2_	0.1013 (ns)	0.6912 (ns)	0.2138 (ns)	0.2659 (ns)	0.1448 (ns)

*PTGS2*					
	**2h**	**4h**	**8h**	**12h**	**18h**
EGF vs Ctrl	0.0197 (^*^)	0.2288 (ns)	0.7446 (ns)	0.5062 (ns)	0.9311 (ns)
PGE_2_ vs Ctrl	0.7688 (ns)	0.028 (^*^)	0.0205 (^*^)	0.1481 (ns)	0.1884 (ns)
EGF+PGE_2_ vs Ctrl	0.009 (^**^)	0.0013 (^**^)	0.0366 (^*^)	0.035 (^*^)	0.6021 (ns)
EGF+PGE_2_ vs EGF	0.2117 (ns)	0.1286 (ns)	0.0881(ns)	0.045 (^*^)	0.7091 (ns)
EGF+PGE_2_ vs PGE_2_	0.0168 (^*^)	0.9534 (ns)	0.6262 (ns)	0.1562 (ns)	0.3235 (ns)

*MYC*					
	**2h**	**4h**	**8h**	**12h**	**18h**
EGF vs Ctrl	0.0441 (^*^)	0.4918 (ns)	0.4346 (ns)	0.3573 (ns)	0.2127 (ns)
PGE_2_ vs Ctrl	0.6387 (ns)	0.0181 (^*^)	0.0648 (ns)	0.1924 (ns)	0.182 (ns)
EGF+PGE_2_ vs Ctrl	0.0099 (^**^)	0.0478 (^*^)	0.0053 (^**^)	0.0038 (^**^)	0.2197 (ns)
EGF+PGE_2_ vs EGF	0.9568 (ns)	0.1388 (ns)	0.02 (^*^)	0.1441 (ns)	0.5973 (ns)
EGF+PGE_2_ vs PGE_2_	0.0112 (^*^)	0.296 (ns)	0.0058 (^**^)	0.1791 (ns)	0.8109 (ns)

*NOS2*					
	**2h**	**4h**	**8h**	**12h**	**18h**
EGF vs Ctrl	0.0448 (^*^)	0.2372 (ns)	0.6838 (ns)	0.4729 (ns)	0.1667 (ns)
PGE_2_ vs Ctrl	0.8677 (ns)	0.0208 (^*^)	0.1401 (ns)	0.265 (ns)	0.5583 (ns)
EGF+PGE_2_ vs Ctrl	0.0478 (^*^)	0.0184 (^*^)	0.0096 (^**^)	0.1155 (ns)	0.0421 (^*^)
EGF+PGE_2_ vs EGF	0.457 (ns)	0.0196 (^*^)	0.0337 (^*^)	0.1283 (ns)	0.0354 (^*^)
EGF+PGE_2_ vs PGE_2_	0.0982 (ns)	0.3008 (ns)	0.1936 (ns)	0.7193 (ns)	0.0835 (ns)

ns = non significant; ^*^ p < 0.05; ^**^ p < 0.01.

**Table 2:** Statistical analysis of nuclear EGFR target genes regulated by EGF and PGE_2_ in EGFR knockout A549 cells bearing EGFR WT

*CCND1*				
	**2h**	**4h**	**8h**	**12h**
EGF vs Ctrl	0.0144(^*^)	0.0369(^*^)	0.7879(ns)	0.1404(ns)
PGE_2_ vs Ctrl	0.5417(ns)	0.0228(^*^)	0.2119(ns)	0.3722(ns)
EGF+PGE_2_ vs Ctrl	0.0220(^*^)	0.0247(^*^)	0.0236(^*^)	0.3111(ns)
EGF+PGE_2_ vs EGF	0.3542(ns)	0.2529(ns)	0.0368(^*^)	0.6618(ns)
EGF+PGE_2_ vs PGE_2_	0.0470(^*^)	0.6414(ns)	0.0497(^*^)	0.8610(ns)

*PTGS2*				
	**2h**	**4h**	**8h**	**12h**
EGF vs Ctrl	0.0162(^*^)	0.0824(ns)	0.9108(ns)	0.2415(ns)
PGE_2_ vs Ctrl	0.9602(ns)	0.0135(^*^)	0.0432(^*^)	0.2181(ns)
EGF+PGE_2_ vs Ctrl	0.0043(^**^)	0.0188(^*^)	0.0179(^*^)	0.2992(ns)
EGF+PGE_2_ vs EGF	0.3345(ns)	0.2700(ns)	0.0387(^*^)	0.9850(ns)
EGF+PGE_2_ vs PGE_2_	0.0150(^*^)	0.4289(ns)	0.0632(ns)	0.9204(^*^)

*MYC*				
	**2h**	**4h**	**8h**	**12h**
EGF vs Ctrl	0.0084(^**^)	0.0593(ns)	0.1278(ns)	0.4770(ns)
PGE_2_ vs Ctrl	0.0169(^*^)	0.0097(^**^)	0.1092(ns)	0.7608(ns)
EGF+PGE_2_ vs Ctrl	0.0156(^*^)	0.0042(^**^)	0.025(^*^)	0.2060(ns)
EGF+PGE_2_ vs EGF	0.4077(ns)	0.0194(^*^)	0.0485(^*^)	0.6039(ns)
EGF+PGE_2_ vs PGE_2_	0.1783(ns)	0.7295(ns)	0.0761(ns)	0.2999(ns)

*NOS2*				
	**2h**	**4h**	**8h**	**12h**
EGF vs Ctrl	0.0222(^*^)	0.1138(ns)	0.4012(ns)	0.4141(ns)
PGE_2_ vs Ctrl	0.4770(ns)	0.0043(^**^)	0.1694(ns)	0.3840(ns)
EGF+PGE_2_ vs Ctrl	0.0151(^*^)	0.0087(^**^)	0.0176(^*^)	0.1467(ns)
EGF+PGE_2_ vs EGF	0.1451(ns)	0.0534(ns)	0.0477(^*^)	0.1423(ns)
EGF+PGE_2_ vs PGE_2_	0.1716(ns)	0.9180(ns)	0.0452(^*^)	0.3600(ns)

ns = non significant; ^*^ p < 0.05; ^**^ p < 0.01.

**Figure 7:**
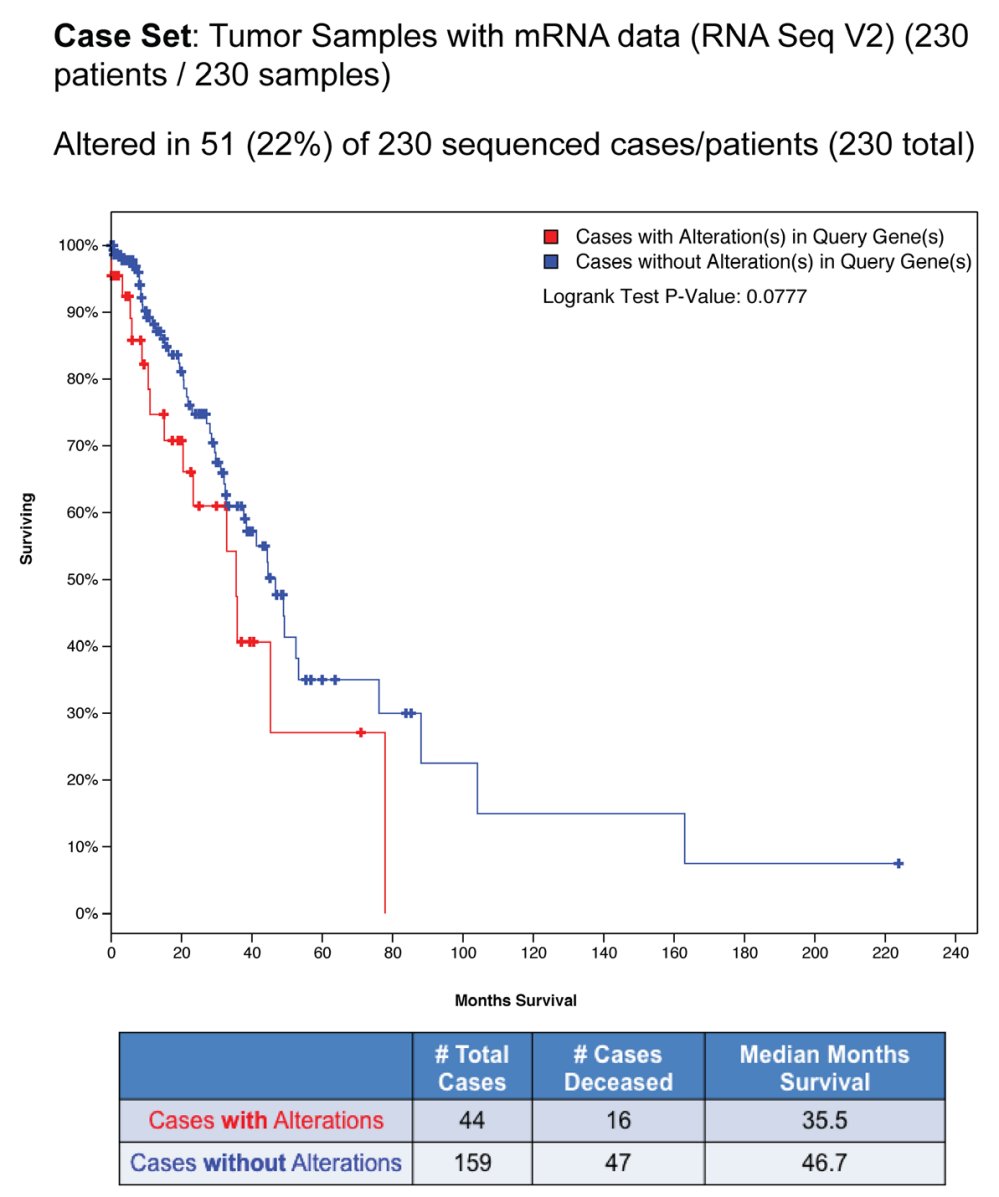
Bioinformatics analysis of nuclear EGFR gene signature in NSCLC patients using cBioPortal database Alterations in nuclear EGFR target genes across TGCA provisional Lung adenocarcinoma samples analyzed by cBioPortal for cancer genomics database. Data were filtered for mRNA upregulation of nuclear EGFR target genes and the percentage on total samples was reported. Overall Survival Kaplan–Meier Estimate curve in lung adenocarcinoma patients with alteration (n= 44) or without alteration (n=159) in nuclear EGFR gene signature (Logrank Test P-Value: 0.0777). The table below the graph summarizes the total cases, deceased cases and median months survival data.

Next, we investigated the prognostic role of the nuclear EGFR gene signature. We exploited the Lung Adenocarcioma TCGA study, including 230 tumor samples with mRNA expression data (RNA Seq V2) by cBioPortal for Cancer Genomics data sets [35, 36]. Alterations, which consists in mRNA upregulation of the nuclear EGFR target genes were found in 51 of 230 patients (22%) as shown in percentages of total samples as follows: *CCND1* (10%)*, PTGS2* (3%)*, MYC* (8%) and *NOS2* (1,7%) (Figure 7). Kaplan–Meier analysis for predicted overall patient survival indicated that cases with alteration of nuclear EGFR gene signature had shorter survival times than those with no alternation (log-Rank test, P < 0.0777), the median survival time being reduced from 46.7 to 35.5 months (Figure 7). Notably, in the group of patients with mRNA upregulation of nuclear EGFR target genes, the deceased patients were 16 on 44 (36.3%), whereas in the group of patients without mRNA upregulation of nuclear EGFR target genes the deceased ones were 47 on 159 (29.5%). These data indicate a trend, even not statistically significant, between nuclear EGFR activity and poor prognosis in lung adenocarcinoma patients.

### EGF and PGE_2_ combination promotes NSCLC cell proliferation

**Figure 8:**
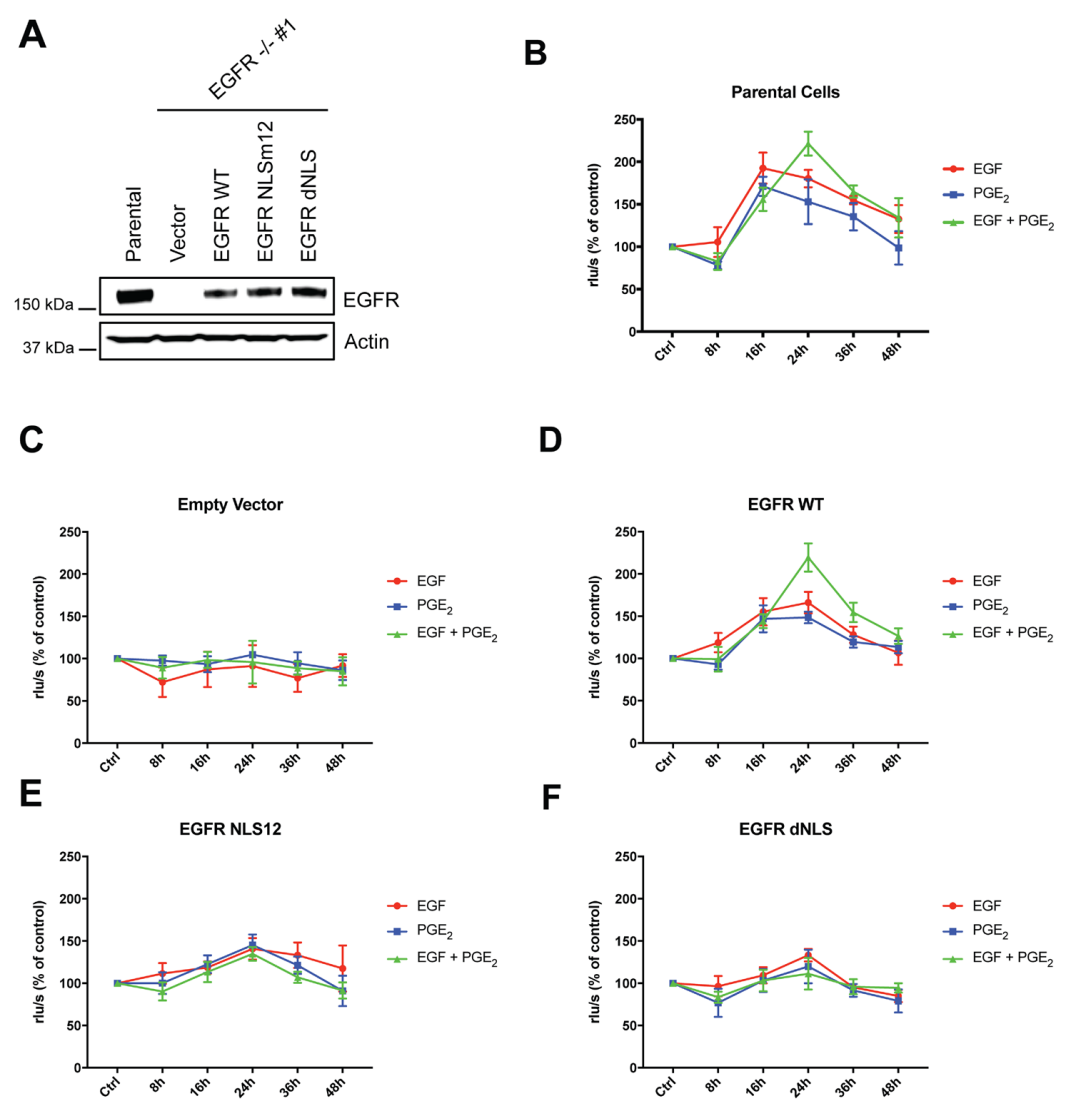
EGF and PGE_2_ induce nuclear EGFR-mediated A549 cell proliferation **(A)** Immunoblotting analysis of EGFR expression in A549 EGFR -/- #1 cells transfected with Vector, EGFR-WT and NLS mutant plasmids for 96 h. Parental cells were included as a control. Actin was used as loading control. **(B–F)** Parental cells or EGFR -/- #1, #2 cells transfected with Vector or EGFR WT or EGFR NLS12 or EGFR dNLS mutant plasmids for 24 h were harvested and seeded for BrdU incorporation assay. Cell proliferation was assessed by measuring the luminescence after 8, 16, 24, 36, 48 h treatment with EGF or PGE_2_ or the combination. Data are mean ± SD of triplicate cultures, expressed as % of control. Statistical analysis is reported in Table 3.

To investigate whether the transcription of nuclear EGFR target genes-induced by the combination of EGF and PGE_2_ could mediate and sustain NSCLC cell proliferation compared to single stimuli, the A549 EGFR-knockout cells described above were used. GLC82 cells knockout for EGFR were generated accordingly and transfected with Empty Vector, or EGFR WT, or NLS mutant plasmids (Supplementary Figure 7A-7B). Similar level of EGFR expression was observed in A549 and GLC82 cells transfected with constructs encoding for WT and mutant (Figure 8A and Supplementary Figure 7C).

Specifically, we assessed whether the disruption of nuclear import of EGFR affected cancer cell proliferation. NSCLC cell proliferation was quantified by using a 5-bromo-2′-deoxy-uridine (BrdU) incorporation assay for 8-48 h (Figure 8B–8F and Supplementary Figure 7D-7H). In parental and EGFR WT expressing A549 cells, EGF and PGE_2_ alone induced cell proliferation in a time-dependent manner with a peak at 16 h, declining toward baseline at 36–48h. In cells exposed to a combination of EGF and PGE_2_, BrdU incorporation levels increased at 16 h, doubled at 24 h and declined to baseline at 48 h, indicating that EGF and PGE_2_ combination-induced nuclear EGFR sustained NSCLC cells growth (Figure 8B–8F). In contrast, cells expressing EGFR-NLS mutants were not comparably stimulated to proliferate in response to neither EGF nor PGE_2_ (Figure 8B–8F). Similar results were obtained in GLC82 cells (Supplementary Figure 7D-7H). Statistical analysis has been reported in Table 3 and Supplementary Table 2 for A549 and GLC82 respectively.

**Table 3:** Statistical analysis of BrdU incorporation assay performed in A549

Parental Cells					
	**8h**	**16h**	**24h**	**36h**	**48h**
EGF vs Ctrl	**0.6082 (ns)**	0.0009 (^***^)	0.0007 (^***^)	0.0005 (^***^)	0.026 (^*^)
PGE_2_ vs Ctrl	0.0012 (^***^)	0.0004 (^***^)	0.0251 (^*^)	0.0187 (^*^)	0.9111 (ns)
EGF+PGE_2_ vs Ctrl	0.0399 (^*^)	0.0021 (^**^)	0.0002 (^***^)	0.0001 (^***^)	0.0627 (ns)
EGF+PGE_2_ vs EGF	0.1207 (ns)	0.0493 (^*^)	0.0146 (^*^)	0.1148 (ns)	0-9314 (ns)
EGF+PGE_2_ vs PGE_2_	0.5137 (ns)	0.208 (ns)	0.0163 (^*^)	0.044 (^*^)	0.1124 (ns)

Empty Vector					
	**8h**	**16h**	**24h**	**36h**	**48h**
EGF vs Ctrl	0.0503 (ns)	0.3501 (ns)	0.5735 (ns)	0.0701 (ns)	0.3463 (ns)
PGE_2_ vs Ctrl	0.5394 (ns)	0.2854 (ns)	0.1136 (ns)	0.5076 (ns)	0.108 (ns)
EGF+PGE_2_ vs Ctrl	0.2124 (ns)	0.774 (ns)	0.7895 (ns)	0.0678 (ns)	0.1904 (ns)
EGF+PGE_2_ vs EGF	0.244 (ns)	0.4509 (ns)	0.8338 (ns)	0.3226 (ns)	0.6124 (ns)
EGF+PGE_2_ vs PGE_2_	0.3548 (ns)	0.5563 (ns)	0.5839 (ns)	0.544 (ns)	0.9156 (ns)

EGFR WT					
	**8h**	**16h**	**24h**	**36h**	**48h**
EGF vs Ctrl	0.0469 (^*^)	0.004 (^**^)	0.0008 (^***^)	0.0073 (^**^)	0.4643 (ns)
PGE_2_ vs Ctrl	0.1155 (ns)	0.007 (^**^)	0.0003 (^***^)	0.0071 (^**^)	0.0334 (^*^)
EGF+PGE_2_ vs Ctrl	0.936 (ns)	0.0007 (^***^)	0.0002 (^***^)	0.0012 (^**^)	0.0073 (^**^)
EGF+PGE_2_ vs EGF	0.1425 (ns)	0.3505 (ns)	0.0116 (^*^)	0.0382 (^*^)	0.1133 (ns)
EGF+PGE_2_ vs PGE_2_	0.5206 (ns)	0.8089 (ns)	0.0024 (^**^)	0.0104 (^*^)	0.1261 (ns)

EGFR NLS12					
	**8h**	**16h**	**24h**	**36h**	**48h**
EGF vs Ctrl	0.1777 (ns)	0.0113 (^*^)	0.0121 (^*^)	0.0189 (^*^)	0.3301 (ns)
PGE_2_ vs Ctrl	0.9847 (ns)	0.0199 (^*^)	0.0183 (^*^)	0.0177 (^*^)	0.4278 (ns)
EGF+PGE_2_ vs Ctrl	0.1889 (ns)	0.1268 (ns)	0.0016 (^**^)	0.1341 (ns)	0.1984 (ns)
EGF+PGE_2_ vs EGF	0.0856 (ns)	0.5404 (ns)	0.1505 (ns)	0.0517 (ns)	0.1943 (ns)
EGF+PGE_2_ vs PGE_2_	0.363 (ns)	0.3641 (ns)	0.1791 (ns)	0.1107 (ns)	0.9641 (ns)

EGFR dNLS					
	**8h**	**16h**	**24h**	**36h**	**48h**
EGF vs Ctrl	0.6342 (ns)	0.1039 (ns)	0.0015 (^**^)	0.043 (^*^)	0.0232 (^*^)
PGE_2_ vs Ctrl	0.116 (ns)	0.0482 (^*^)	0.1585 (ns)	0.1676 (ns)	0.1562 (ns)
EGF+PGE_2_ vs Ctrl	0.0122 (^*^)	0.6773 (ns)	0.352 (ns)	0.4671 (ns)	0.1562 (ns)
EGF+PGE_2_ vs EGF	0.1783 (ns)	0.5399 (ns)	0.1325 (ns)	0.9016 (ns)	0.1454 (ns)
EGF+PGE_2_ vs PGE_2_	0.7811 (ns)	0.4621 (ns)	0.1999 (ns)	0.7544 (ns)	0.0531 (ns)

ns = non significant; ^*^ p < 0.05; ^**^ p < 0.01; ^***^ p < 0.001.

**Figure 9:**
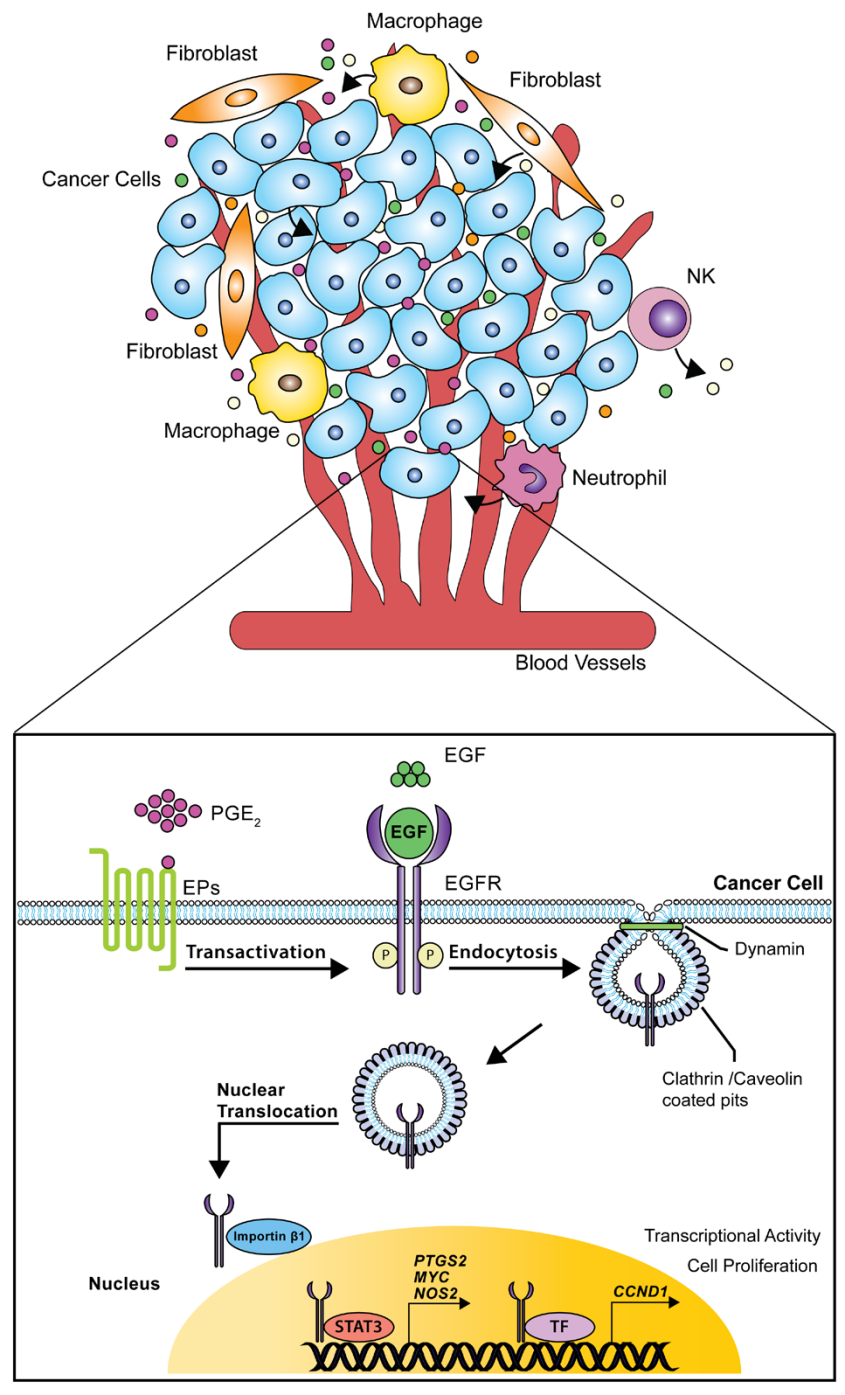
Schematic model of nuclear EGFR signaling in NSCLC cells The tumor microenvironment consists in cancer cells surrounded by different cell types such as stromal, endothelial, inflammatory and immune cells. In this scenario, single entities release mediators, cytokines and growth factors thereby supporting tumor progression. Particularly, EGF and PGE_2_ activate or transactivate EGFR leading to its internalization via dynamin-dependent Clathrin and Caveolin endocytosis. Further, Importin β1 transports the NLS-bearing EGFR across the nuclear envelope to the nucleoplasm. Within the nucleus, EGFR interacts with STAT3 or other transcription factors (TF) promoting the transcription of *CCND1*, *PTGS2*, *MYC* and *NOS*, a process that culminates with increased tumor cell proliferation.

In summary, we have identified the mechanisms by which PGE_2_ promotes EGFR nuclear translocation in human NSCLC cells. PGE_2_ internalizes EGFR through Clathrin and Caveolin- mediated endocytosis, whereas EGF preferentially promotes Clathrin-mediated EGFR internalization. Inside the cell, EGFR is imported to the nucleus by Importin β1 and associates with activated STAT3, and potentially other transcription factors, to induce the expression of nuclear EGFR target genes, thereby promoting cancer cell proliferation (Figure 9). The gene signature linked to EGFR nuclear internalization in NSCLC cell lines matches that of patients with poor prognosis observed in patient with early stage NSCLC [37], suggesting a potential correlation with the clinical setting.

## DISCUSSION

The inflammatory mediator PGE_2_ is known to favor growth of several epithelial tumor cells in which the oncogenic drive is sustained by EGF/EGFR system. Two mechanisms are recognized as inducers of EGFR-mediated oncogenicity. The first one, occurring predominately at the cell membrane, consists in the activation of the receptor followed by a well-known effector cascade [38]. The second one involves EGFR internalization from the plasma membrane to the nucleus in which it serves as co-transcriptional factor [29, 39]. Recently, we dissected the mechanisms of PGE_2_-mediated EGFR nuclear translocation showing marked differences in the kinetic and in the internalization pattern between EGF and PGE_2_ [12]. Here, we tested whether different endocytic mechanisms are involved in EGF and PGE_2_-mediated EGFR nuclear translocation. Based on differences in time of induction and the internalization patterns, we hypothesized distinct mechanisms promoted by EGF or PGE_2_.

EGFR is mainly internalized via Clathrin-mediated endocytosis [16] nevertheless, saturation of Clathrin or stimulation with different ligands could trigger alternative routes of internalization, including Caveolin-mediated endocytosis and macropinocytosis [17–19]. Using pharmacological inhibitors and siRNA targeting Clathrin heavy chain and Caveolin-1, we found that Clathrin knockdown abrogated EGFR nuclear translocation upon either EGF or PGE_2_ treatment. However, Caveolin-1 knockdown or pharmacological inhibition (methyl-β-cyclodextrin (MβCD) suppressed solely PGE_2_-mediated EGFR internalization (Figures 1–2, Supplementary Figures 1–2). These findings demonstrate that Clathrin has a key role in EGFR internalization, whereas PGE_2_ uses Caveolin as an alternative endocytic route to shuttle EGFR from the cell membrane to the nucleus. Notably, the uptake kinetics of Caveolin-mediated endocytosis is slower than that of Clathrin [40, 41], an observation that may explain the differences in the kinetics between EGF and PGE_2_-mediated EGFR nuclear import. Moreover, we do not observe any enhancement in the modulation of PGE_2_ activity on nuclear EGFR translocation when NSCLC cells were silenced for Clathrin heavy chain and Caveolin-1 together, suggesting that the two endocytic pathways activated by the prostanoid work independently (Supplementary Figure 3). However, we may not exclude that due to technical limitation, we could not discern the quantitative contribution of each individual endocytic routes.

As nuclear import of proteins bearing nuclear localization sequence (NLS) requires the interaction with Importin α/β for nuclear internalization [11], we show the key role of Importin β1 in PGE_2_-mediated EGFR nuclear translocation, as already reported for EGF (Figure 3).

Transcriptional regulation of genes involved in cell proliferation is one of the main functions of nuclear EGFR [39]. EGFR lacks a DNA binding domain and exerts its co-transcriptional functions via association with several transcription factors, including STAT3, STAT5 and E2F1 [42]. Among nuclear EGFR target genes, previously shown to be modulated by PGE_2_ in NSCLC cells, *PTGS2*, *MYC* and *NOS2* have been shown to be induced by the EGFR-STAT3 complex [12, 31, 32, 34].

Based on these notions, we investigated whether PGE_2_ leads to STAT3 activation and we found that the prostainoid promotes STAT3 phosphorylation with a peak at 30 min (Figure 4A). Upstream to STAT3 activation mediated by PGE_2_, JAK activity appears to be required, as its inhibition significantly reduced PGE_2_-mediated activity (Figure 4B). We have assessed a direct EGFR-STAT3 interaction by co-immunoprecipation and find that STAT3, in both non-phosphorylated and phosphorylated conditions, physically associates and colocalizes with EGFR following PGE_2_ treatment (Figure 4C–4E). Moreover, Caveolin-1 knockdown affected PGE_2_-mediated EGFR-STAT3 association, highlighting the functional role of Caveolin endocytosis in PGE_2_ activity. (Figure 4F–4G and Supplementary Figures 4 and 5).

By analyzing the expression of target genes of nuclear EGFR in NSCLC cells, we found that *CCND1*, *PTGS2*, *MYC* and *NOS2* mRNA levels were upregulated by EGF as well as by PGE_2_, yet with different kinetics indicating the involvement of distinct internalization pathways (Figure 6 and Supplementary Figure 6). Interestingly, the combination of treatments induced a prolonged transcriptional activation of nuclear EGFR gene signature compared to individual stimuli (from 2-4 to 8h), indicating that the PGE_2_ and EGF cooperate in their activities to sustain tumor progression. Additionally, by using cBioPortal for Cancer Genomics database, we have found a reduction in median months of survival in lung cancer patients that exhibit an upregulation of a nuclear EGFR gene signature, thus showing a trend between nuclear EGFR and poor prognosis in NSCLC (Figure 7). It is worth to mention that these data were derived from a non-selected patient population characterized by large differences in: age, sex, tumor stage, smoking history and therapy, therefore representing a heterogeneous situation. Among nuclear EGFR target genes, *MYC* is of high prognostic value in different epithelial tumors [43], and its expression is driven by PGE_2_ in NSLC cells [44]. Further, *MYC* appears to be one of the most amplified genes across nuclear EGFR transcriptional signature, further corroborating our findings.

To demonstrate that tumor gene reprogramming promoted by EGF and PGE_2_-induced nuclear EGFR is critical for tumor progression, we have assessed cell proliferation by BrdU incorporation assay in NSCLC cells. A549 and GLC82 EGFR knockout cells expressing wild type EGFR increase in their proliferation in response to PGE_2_ or EGF in contrast to cells transfected with mutant EGFR into its nuclear localization sequence (Figure 8B–8F and Supplementary Figure 7D-7H). Notably, the combined treatment potentiates nuclear EGFR mitogenic activity in terms of time and intensity, supporting the hypothesis of EGF and PGE_2_ malignant alliance.

In this scenario, we identified PGE_2_ as an inducer of EGFR nuclear translocation in NSCLC cells, an event, which has clinical correlate with poor prognosis [44, 45]. PGE_2_ coupling with EP receptor triggers EGFR internalization. In particular, we showed that EGFR undergoes to Clathrin- and Caveolin-mediated endocytosis leading to association with Importin β1 and consequent nuclear import. Within the nucleus, EGFR interacts with transcription factors such as STAT3 to regulate gene expression. Particularly, nuclear EGFR induced by EGF or PGE_2_ or the combination of treatments, promotes *CCND1*, *PTGS2*, *MYC* and *NOS2* upregulation and sustains tumor growth (Figure 9), supporting the hypothesis that the gene signature associated with nuclear EGFR is correlated with poor prognosis in NSCLC and may serve as a biomarker for patients outcome and treatment selection.

Collectively, the findings of this work on EGFR nuclear import and gene transcription, assume the character of a self-feeding loop which leads to persistent up-regulation of *CCND1*, *PTGS2*, *NOS2* and *MYC*, hence overproduction of PGE_2_, NO and overload of MYC which support a favorable pro-tumor microenvironment.

## MATERIALS AND METHODS

### Cell culture and cultured conditions

The human NSCLC cancer cell line A549 (CCL-185), was purchased from American Type Culture Collection and the GLC82 NSCLC cell line was kindly provided by Dr. Mario Chiariello (Istituto Toscano Tumori, Siena, Italy). Cells were certified by STRA, (LGC Standards S.r.l., Sesto San Giovanni, Milan, Italy) and were cultured in DMEM for A549 and in RPMI-1640 (Euroclone, Milan, Italy) for GLC82 supplemented with 10% FBS and 2 mM Glutamine, 100 Units Penicillin and 0.1mg/l Streptomycin (Sigma Aldrich, St. Louis, MO, USA) in a humidified incubator with 5% CO_2_ at 37°C. A549 and GLC82 were immediately expanded after delivery (up to 6 × 10^7^ cells) and frozen down (1 × 10^6^ per vial) such that both cell lines could be restarted after a maximum of 10 passages every 3 months from a frozen vial of the same batch of cells. Control of mycoplasma was performed regularly using MycoAlert™ PLUS Mycoplasma Decection Kit (#LT07-710 Lonza, Basel, Switzerland).

### Chemicals and reagents

Recombinant human EGF (#AF-100-15) was purchased from PeproTech (Rocky Hill, NJ, USA). PGE_2_ (#P0409), Dynasore (#D7693), 5-N-Ethyl-N-isopropyl amiloride (EIPA) (#A3085) and Metil beta cyclodextrin (#C4555,) were purchased from Sigma Aldrich. Ruxolitinib (#S1378) was purchased from Selleckchem (Houston, TX, USA). STAT3 Inhibitor VII Calbiochem (#573103) was purchased from Merck Millipore (Darmstadt, Germany).

### Antibodies

Anti-EGFR (#4267), anti-STAT3 (#9139), anti-pSTAT3 Tyr 705 (#9138), anti-Caveolin-1 (#3267), anti-Clathrin Heavy Chain (#4796), anti-Importin-β1 (#8673) antibodies were purchased from Cell Signaling Technology (Danvers, MA, USA). Anti-Tubulin antibody (#T9026) was purchased from Santa Cruz (Heidelberg, Germany). Anti-Lamin A (#SAB4501764), anti-Actin (#A5441) antibodies were obtained from Sigma Aldrich.

### Whole cell extracts

Cells were washed 2x with cold Dulbecco’s Phosphate Buffered Saline (Sigma Aldrich) and lysed on ice with CelLytic™ MT Cell Lysis Reagent (#C3228 Sigma Aldrich) supplemented with 2mM Na_3_VO_4_ and 1x Protease inhibitor cocktail for mammalian cells (#P2714 Sigma Aldrich). Cell lysates were centrifuged at 16000 × g for 20 min at 4°C and the supernatants were then collected for immunoblot.

### Cell fractionation

Nuclear and cytoplasmic extracts were prepared with NE-PER™ (#78833) nuclear and cytoplasmic extraction reagents (ThermoFisher Scientific, Waltham, MA, USA) following the manufacturer’s instructions as described previously [11].

### Immunoblotting analysis

4 × 10^5^ cells were plated in 60 mm dishes, serum deprived (0.1%. fetal calf serum, overnight), then treated as described in the text. Protein concentration was determined using the BCA protein assay kit (#23227 ThermoFisher Scientific). For whole cell lysates, an equal amount of proteins were loaded on SDS–polyacrylamide gel and then transferred to a nitrocellulose membrane (#10600002 GE Healthcare Lifesciences) For cell fractionation experiments, 10μg of the cytosolic and 40μg of the nuclear extracts were applied for SDS-PAGE. Immunoblot analysis was performed as described previously [8, 10]. Signals were detected by SuperSignal WestPico Chemiluminescent Substrate (#34578 ThermoFisher Scientific) using ChemiDoc system and Quantity one software (Bio-Rad, Hercules, CA, USA). All experiments were performed at least three times. For all experiments using whole cell lysate, Actin was used as loading control. Lamin-A and Tubulin were used as loading and purity controls for the nuclear and cytosolic fractions, respectively. Immunoblots were analyzed by densitometry using NIH Image J 1.48v software, and the results, expressed as arbitrary density units (A.D.U.) ±SD, were normalized to Actin, EGFR, STAT3, Lamin-A or Tubulin.

### Immunofluorescence microscopy analysis

Cells were plated on glass coverslips, starved overnight and treated as described in the text. Cells were fixed using 4% paraformaldehyde/PBS for 15 min and permeabilized with 0.5% NP-40 for 5 min. Next, cells were blocked using 3% BSA, 0.01% TritonX-100 in PBS for 20 min. Furthermore, cells were incubated with anti-EGFR, overnight at 4°C followed by 3x washes with PBS/ 0.01% TritonX-100 and incubation with the AlexaFluor^®^ 488-labeled (#A11034 Invitrogen, Carlsbad, CA, USA) for 60 min at room temperature. After 3x washes with PBS/ 0.01% TritonX-100, nuclei were stained with 6-diamidino-2- phenylindole (DAPI #D9542) or propidium iodide (PI #P4864) 1μg/ml (Sigma Aldrich) for 20 min. The coverslips were mounted with fluorescent mounting medium (#S3023 Dako, Glostrup, Denmark) on microscope slides. Cells were analyzed with a confocal laser scanning microscope Leica SP5. Images for documenting EGFR nuclear translocation and colocalization with Caveolin-1 were acquired in the middle section of the nuclei with 63x magnification.

### Transfection of siRNAs and plasmids

siRNAs used for transient knock-down experiments were purchased from Qiagen (Hilden, Germany). Cells were transfected with 20nM targeting siRNA or scrambled control siRNA using Lipofectamine^®^ RNAiMAX (#13778150 Invitrogen) according to manufacturer’s instructions. Cells were assayed 48-72h after transfection. Knockdown efficiency was assessed by immunoblotting. Target sequences are listed in Supplementary Table 3.

For DNA transfection, cells were transfected with 1–10 μg plasmid using Lipofectamine^®^ 2000 (#12566014 Invitrogen) according to manufacturer’s instructions. EGFR WT and NLS mutant plasmids (NLSm12 and dNLS) were kindly provided by Prof. Mien-Chie Hung (University of Texas MD Anderson Cancer Center, Houston, TX, USA) [32]. pSpCas9(BB)-2A-GFP (PX458) (#48138) was purchased from Addgene. Cells were analyzed 24–96 h post-transfection.

### Knockout of EGFR by CRISPR/Cas9-mediated genome editing

A549 and GLC82 EGFR knockout cells were generated by CRISPR/Cas9 as previously described [12].

### Immunoprecipitation

1 × 10^6^ were plated in 100 mm diameter dishes and starved overnight in 0.1% FBS. Then, cells were treated according to experimental design and lysed with CelLytic™ MT Cell Lysis Reagent (Sigma Aldrich) or subjected to cell fractionation with NE-PER™ nuclear and cytoplasmic extraction reagents (ThermoFisher Scientific). 300 μg of total proteins per sample were immunoprecipitated using Dynabeads™ Protein G Immunoprecipitation Kit (#10007D ThermoFisher Scientific) with an Anti-EGFR antibody (#4267 Cell signaling) following the manufacturer’s instructions. Immunoprecipitates were solubilized in LDS Sample Buffer (#NP0007 ThermoFisher Scientific) supplemented with Sample reducing agent (#NP0004 ThermoFisher Scientific), boiled for 10 min, separated in SDS/4-12% polyacrylamide gel and transferred onto nitrocellulose membranes. Immunoblotting analysis was performed using anti-STAT3 (#9139 Cell signaling) or anti-pSTAT3 Tyr 705 (#9138 Cell signaling) antibodies.

### Real time PCR

Total RNA was prepared using RNeasy Plus Kit (#74134 Qiagen) following manufacturer’s instructions. 1 μg RNA was reverse transcribed using QuantiTect Reverse Transcription Kit (# 205313 Qiagen) and quantitative RT-PCR was performed using QuantiNova SYBR Green PCR Kit (#208056 Qiagen) in a Rotor-Gene Q PCR machine (Qiagen). Fold change expression was determined by the comparative Ct method (ΔΔCt) normalized to 60S Ribosomal protein L19 expression. qRT-PCR data are represented as fold increase relative to non-treated cells (Control), which were assigned to 1. Primers for quantitative RT-PCR are listed in Supplementary Table 4.

### Analysis of nuclear EGFR gene signature in TGCA provisional lung adenocarcinoma samples

cBioPortal for Cancer Genomics dataset (http://cbioportal.org) was used to analyze the nuclear EGFR target gene signature, which consists of *CCND1*, *PTGS2*, *MYC* and *NOS2* mRNA upregulation in lung adenocarcinoma TCGA study that includes 230 sequenced samples [35, 36]. The summary of mRNA upregulation of nuclear EGFR target genes across tumor samples reported in the text was generated using OncoPrint view. The available survival data are displayed as Kaplan-Meier plots with P values derived from a logrank test.

### BrdU incorporation assay

Cell proliferation was determined by 5-bromo-2′-deoxy-uridine (BrdU) incorporation using a chemioluminescence ELISA according to the manufacturer’s instructions (#11669915001 Roche Diagnostic S.p.A, Monza, Italy). Briefly, 3 × 10^3^ cells were seeded in 96-well plate and starved overnight in growth medium 0.1% FBS. Next, cells were exposed to 5ng/ml EGF or 1μM PGE_2_ or combination of treatments for 8, 16, 24, 36 and 48 h. BrdU was added during the late stage (8 h) of incubation. Then, cells were processed following manufacturer’s protocol. Chemiluminescence generated by BrdU labelled cells was measured using Infinite F200 Pro luminometer (Tecan life sciences, Switzerland). Data are reported as % relative to non-treated cells.

### Statistical analysis

Statistical analysis and graphs were generated using the GraphPad Prism software (San Diego, CA, USA). All statistical analysis was done by unpaired/paired Student’s *t*-test, p-value < 0.05 was considered significant.

## SUPPLEMENTARY MATERIALS FIGURES AND TABLES



## Abbreviations

A.D.U: Arbitrary density unit
ADAM: A disintegrin and metalloproteinase
BrdU: 5-bromo-2′-deoxy-uridine
CCND1: Cyclin D1
CIE: Clathrin-independent endocytosis
CME: Clathrin-mediated endocytosis
DAPI: 4′,6-diamidino-2-phenylindole
DYN: Dynasore
E2F1: Retinoblastoma-Associated Protein 1
EGF: Epidermal growth factor
EGFR: Epidermal growth factor receptor
EIPA: 5-N-Ethyl-N-isopropyl amiloride
JAK: Janus Kinase
MYC: c-Myc
MβCD: Methyl-β-cyclodextrin
NLS: Nuclear localization sequence
NOS2: Inducible Nitric oxide synthase
NSCLC: Non-small cell lung cancer
PGE2: Prostaglandin E2
PI3K: Phosphatidylinositol-4,5-Bisphosphate 3-Kinase
PKA: Protein kinase A
PKC: Protein kinase C
PTGS2: Cyclooxygenase 2
qRT-PCR: Quantitative Real Time PCR
SD: Standard deviation
siRNA: Small interfering RNA
SRC: V-Src Avian Sarcoma (Schmidt-Ruppin A-2) Viral Oncogene
STAT3: Signal transducer and activator of transcription 3
STAT5: Signal transducer and activator of transcription 5
TCGA: The cancer genome atlas
WT: Wild Type
