# Supplementary material for: PGE2 mediates EGFR internalization and nuclear translocation via caveolin endocytosis promoting its transcriptional activity and proliferation in human NSCLC cells
Source: Oncotarget. 2018 Feb 15;9(19):14939–58. doi: 10.18632/oncotarget.24499 (PMC5871088; doi:10.18632/oncotarget.24499)
Supplement: Supplementary file 1 [file oncotarget-09-14939-s001.pdf]

PGE2 mediates EGFR internalization and nuclear translocation *via* caveolin endocytosis promoting its transcriptional activity and proliferation in human NSCLC cells

SUPPLEMENTARY MATERIALS

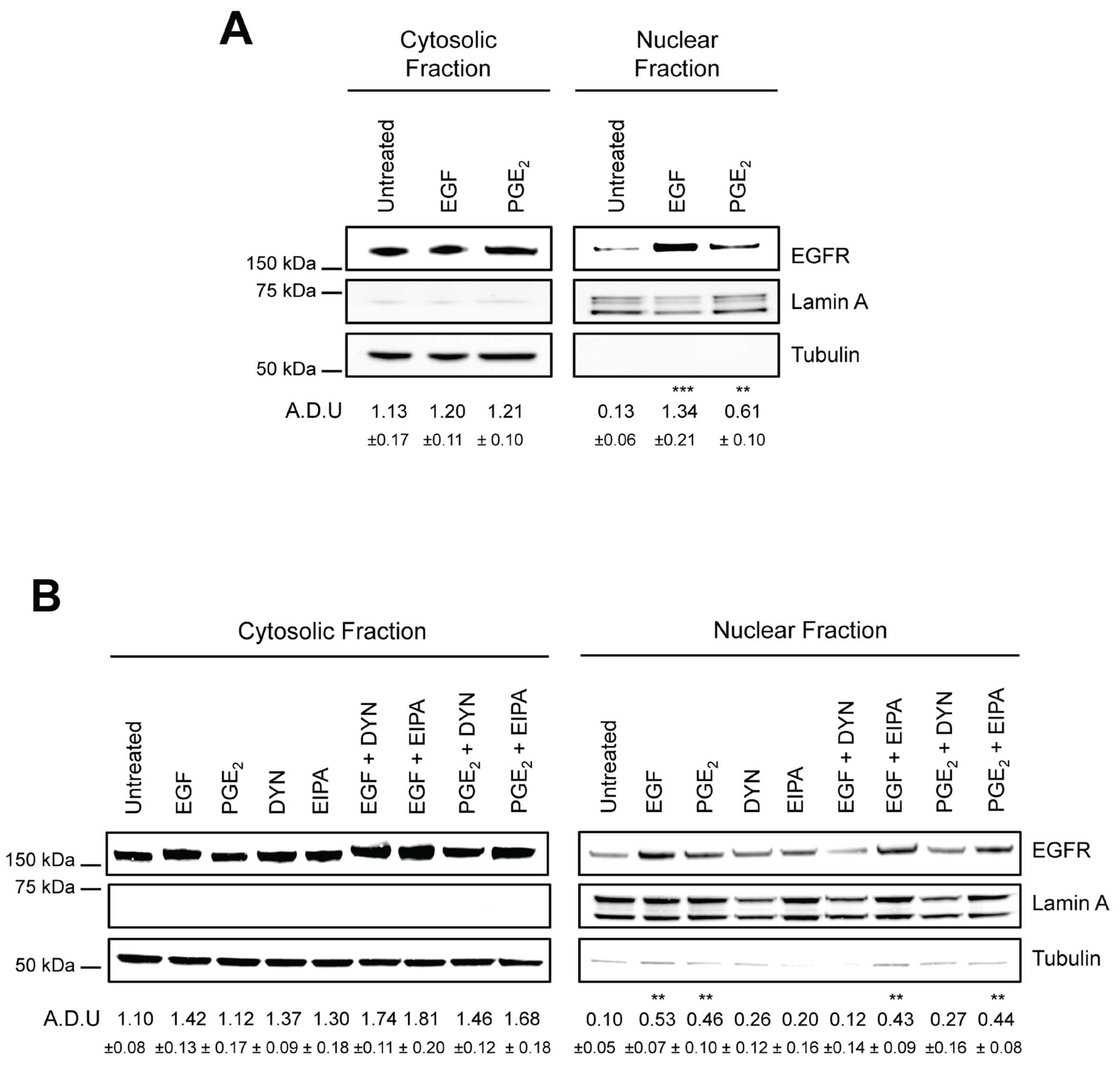

Supplementary Figure 1: Dynamin inhibition blocks EGF- and PGE_2-induced EGFR nuclear translocation in GLC82. **(A)** Immunoblotting analysis of EGFR expression in cytosolic and nuclear fraction in overnight starved GLC82 exposed to 10 min to 25ng/ml EGF or 1µM PGE_2. **(B)** GLC82 cells were starved overnight and then treated with dynasore 80µM (DYN) or 100µM 5-(N-Ethyl-N-isopropyl) amiloride (EIPA) for 30 min before challenge with 25 ng/ml EGF or 1µM PGE_2 for 10 and 60 min respectively. EGFR level in cytoplasmic and nuclear fraction was assessed using immunoblot with indicated antibodies. Tubulin and Lamin A were used as loading control for cytosolic and nuclear fraction. Immunoblotting quantification was expressed in A.D.U. (arbitrary density unit) and as mean ± SD. $^{*}p < 0.05$, $^{**}p < 0.01$, $^{***}p < 0.001$ vs Ctrl. EGFR in the cytoplasmic and nuclear fractions was normalized to Tubulin or Lamin A respectively. The experiments were performed three times.

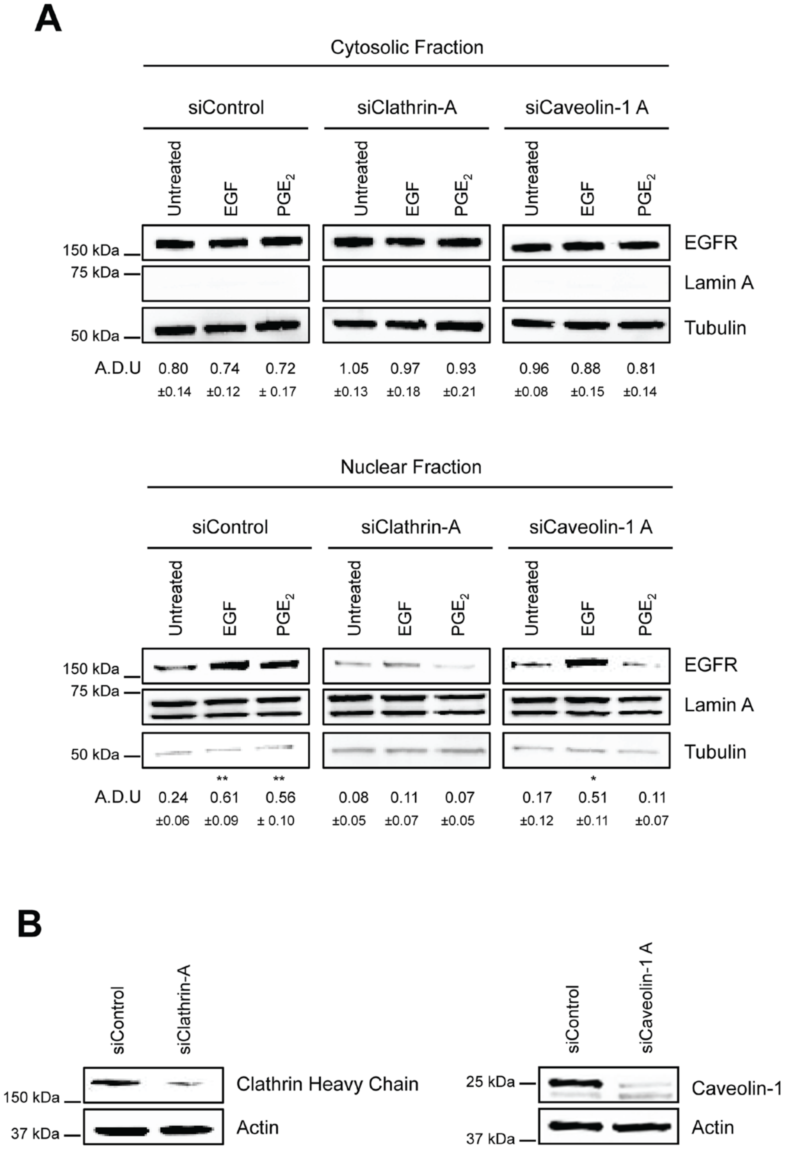

Supplementary Figure 2: PGE_2 promotes EGFR internalization via Clathrin- and Caveolin-mediated endocytosis in GLC82. **(A)** GLC82 cells were transfected with siRNA control or siRNAs against Clathrin Heavy Chain or Caveolin-1 for 24 h. After that, cells were serum starved overnight and then exposed to 25ng/ml EGF for 10 min or to 1μM PGE_2 for 60 min. EGFR level in cytoplasmic and nuclear fraction was assessed using immunoblot with indicated antibodies. Tubulin and Lamin A were used as loading control for cytosolic and nuclear fraction. Immunoblotting quantification was expressed in A.D.U. (arbitrary density unit) and as mean ± SD. $^{*}p < 0.05$, $^{**}p < 0.01$ vs Ctrl. EGFR in the cytoplasmic and nuclear fractions was normalized to Tubulin or Lamin A respectively. **(B)** Knockdown efficiency was verified by immunoblotting with Clathrin Heavy Chain or Caveolin-1 antibodies, actin was used as loading control. The experiments were performed three times.

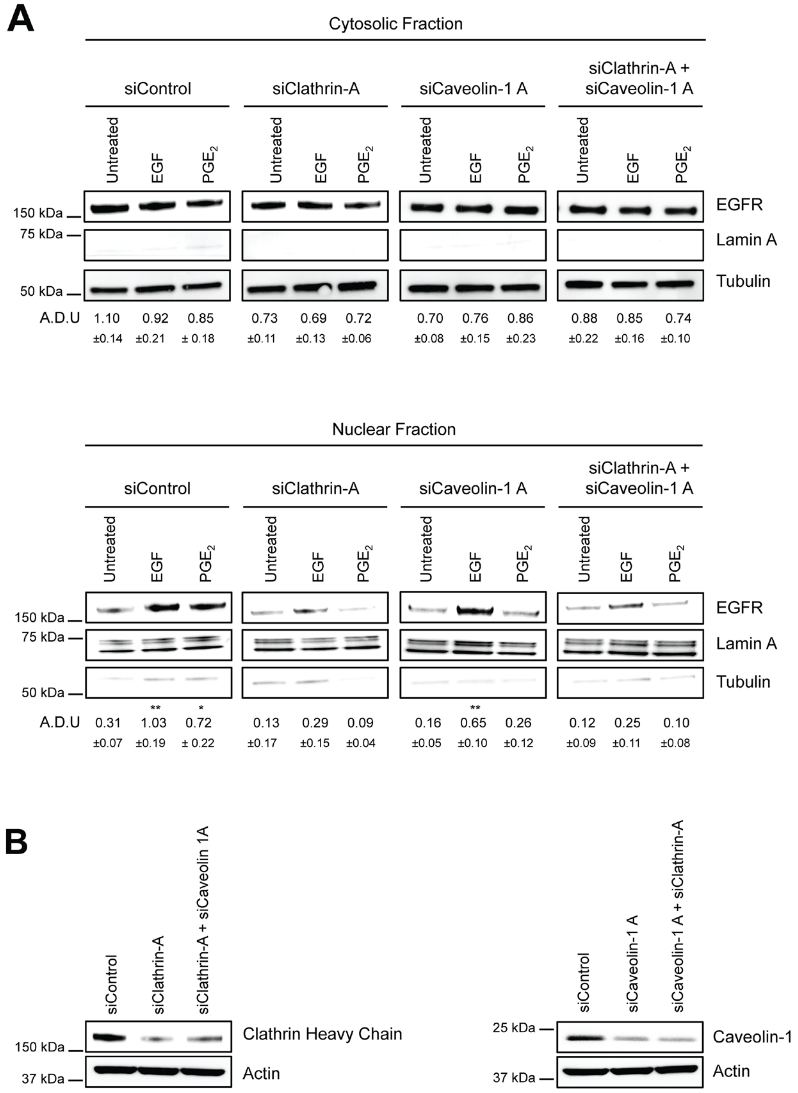

Supplementary Figure 3: Concurrent knockdown of Clathirin Heavy Chain and Caveolin-1 did not enhance the inhibition of PGE_2-induced EGFR nuclear translocation compared to individual siRNAs. **(A)** A549 cells were transfected with siRNA control or siRNAs against Clathrin Heavy Chain or Caveolin-1 or the combination for 24 h. After that, cells were serum starved overnight and then exposed to 25ng/ml EGF for 10 min or to 1μM PGE_2 for 60 min. EGFR level in cytoplasmic and nuclear fraction was assessed using immunoblot with indicated antibodies. Tubulin and Lamin A were used as loading control for cytosolic and nuclear fraction. Data are shown only for siClathrin-A and siCaveolin-1A, similar data were obtained with siClathrin-B, siCaveolin-1B and siClathrin-B+ siCaveolin-1B. Immunoblotting quantification was expressed in A.D.U. (arbitrary density unit) and as mean ± SD. $^{*}p < 0.05$, $^{**}p < 0.01$ vs Ctrl. EGFR in the cytoplasmic and nuclear fractions was normalized to Tubulin or Lamin A respectively. **(B)** Knockdown efficiency was verified by immunoblotting with Clathrin Heavy Chain or Caveolin-1 antibodies, actin was used as loading control. The experiments were performed three times.

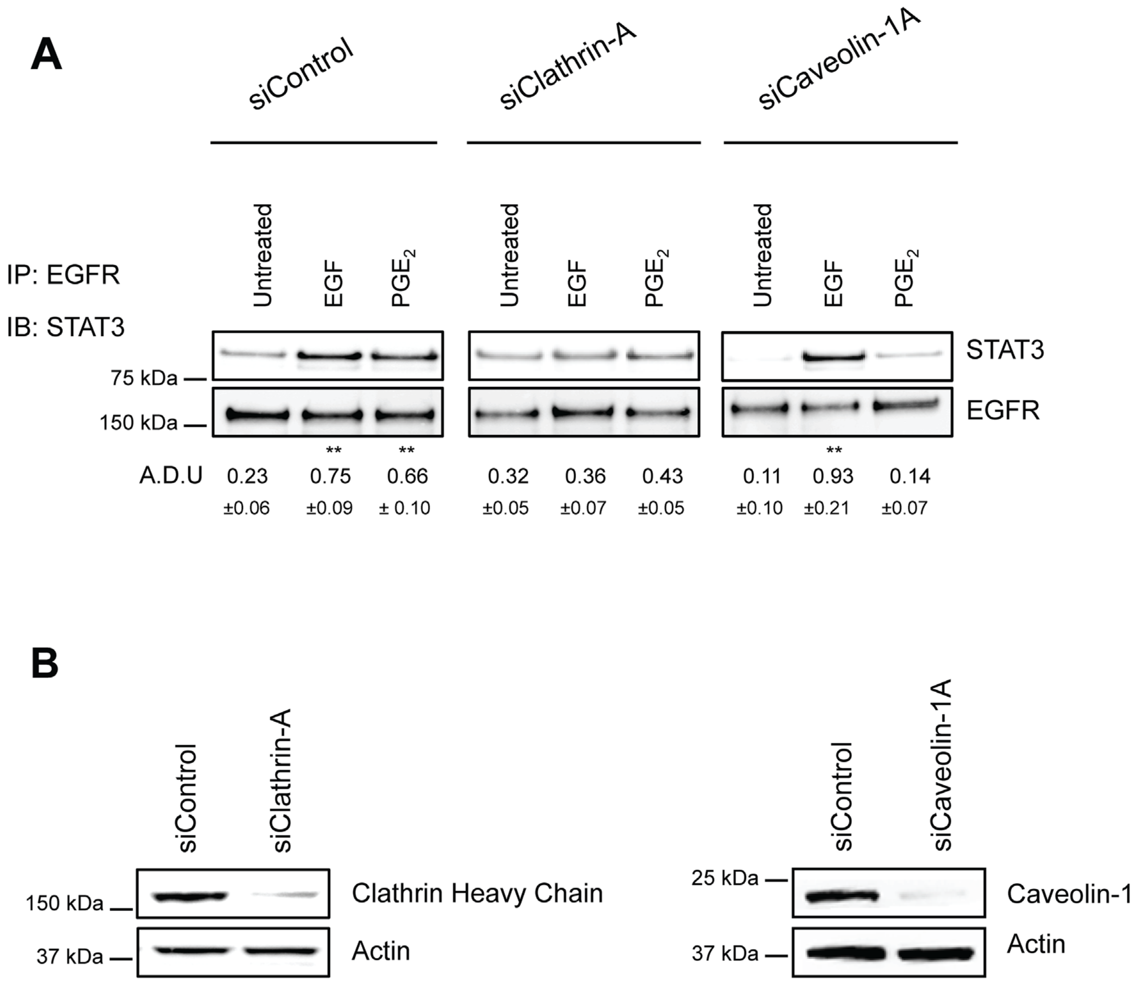

Supplementary Figure 4: PGE_2 induces the formation of EGFR-STAT3 complex into the nucleus in GLC82. (A) GLC82 cells were transfected with siRNA Control or siRNA against Clathrin Heavy Chain or against Caveolin-1 for 24 h. After that, cells were serum starved overnight and treated with 25ng/ml EGF for 10 min or 1μM PGE_2 for 60 min. Whole cell lysates were subjected to immunoprecipitation with anti-EGFR antibody and analyzed by immunoblotting with anti-STAT3 antibody. Immunoblotting quantification was expressed in A.D.U. (arbitrary density unit) and as mean ± SD. $^{**}p < 0.01$ vs Ctrl. STAT3 was normalized to EGFR. **(B)** Knockdown efficiency was verified via western blot with Clathrin heavy chain and Caveolin-1 antibodies, actin was used as loading control. The experiments were performed three times.

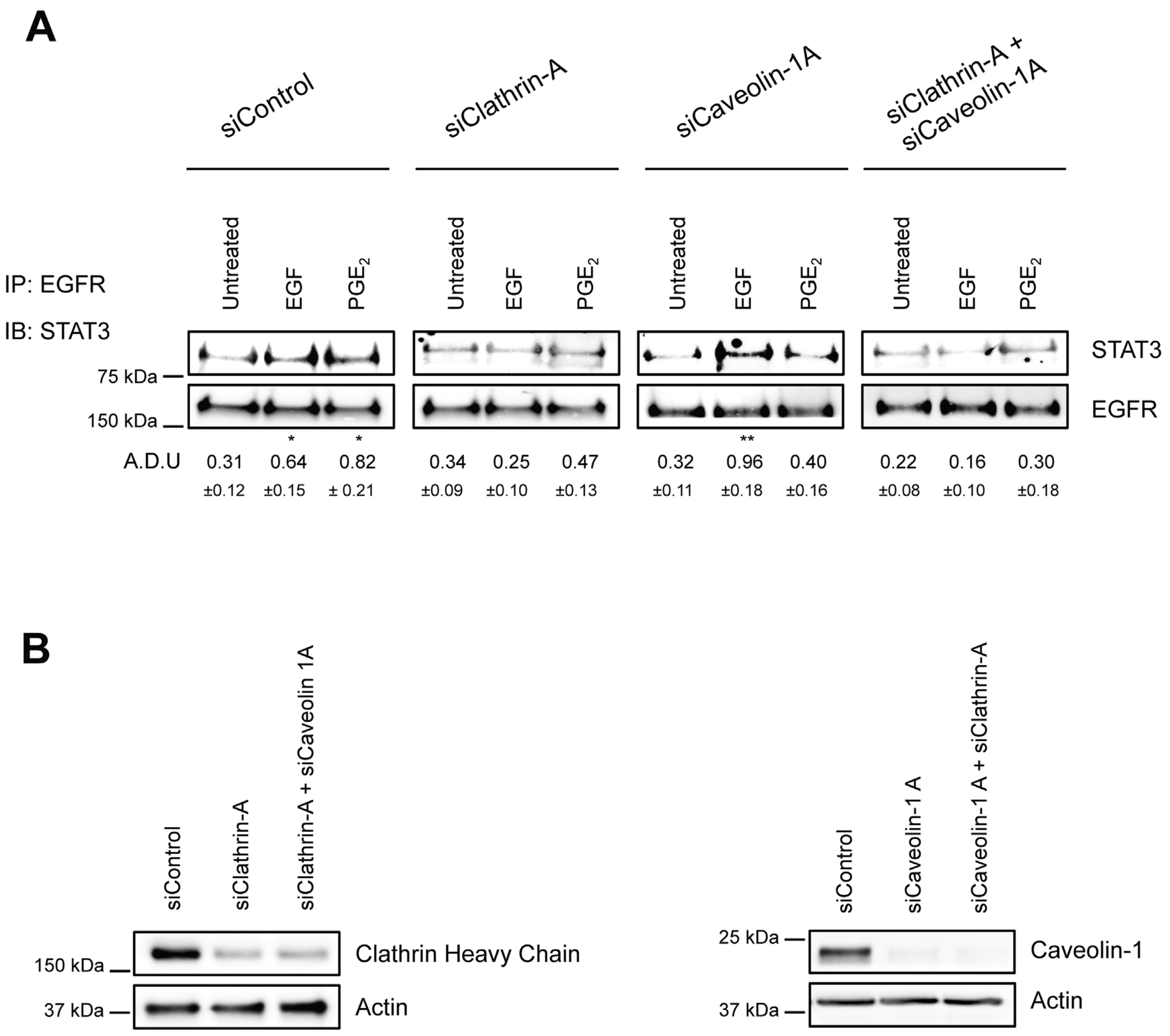

Supplementary Figure 5: Concurrent knockdown of Clathirin Heavy Chain and Caveolin-1 did not enhance the inhibition of PGE_2-induced EGFR-STAT3 co-immunoprecipitation compared to individual siRNAs. **(A)** A549 cells were transfected with siRNA Control or siRNA against Clathrin Heavy Chain or against Caveolin-1 or the combination for 24 h. Cells were then serum starved overnight and treated with 25ng/ml EGF for 10 min or 1μM PGE_2 for 60 min. Whole cell lysates were subjected to immunoprecipitation with anti-EGFR antibody and analyzed by immunoblotting with anti-STAT3 antibody. **(B)** Knockdown efficiency was verified via western blot with Clathrin heavy chain and Caveolin-1 antibodies, actin was used as loading control. Data are shown only for siClathrin-A and siCaveolin-1A, similar data were obtained with siClathrin-B, siCaveolin-1B and siClathrin-B+ siCaveolin-1B. Immunoblotting quantification was expressed in A.D.U. (arbitrary density unit) and as mean ± SD. $^{*}p < 0.05$, $^{**}p < 0.01$vs Ctrl. STAT3 was normalized to EGFR. The experiments were performed three times.

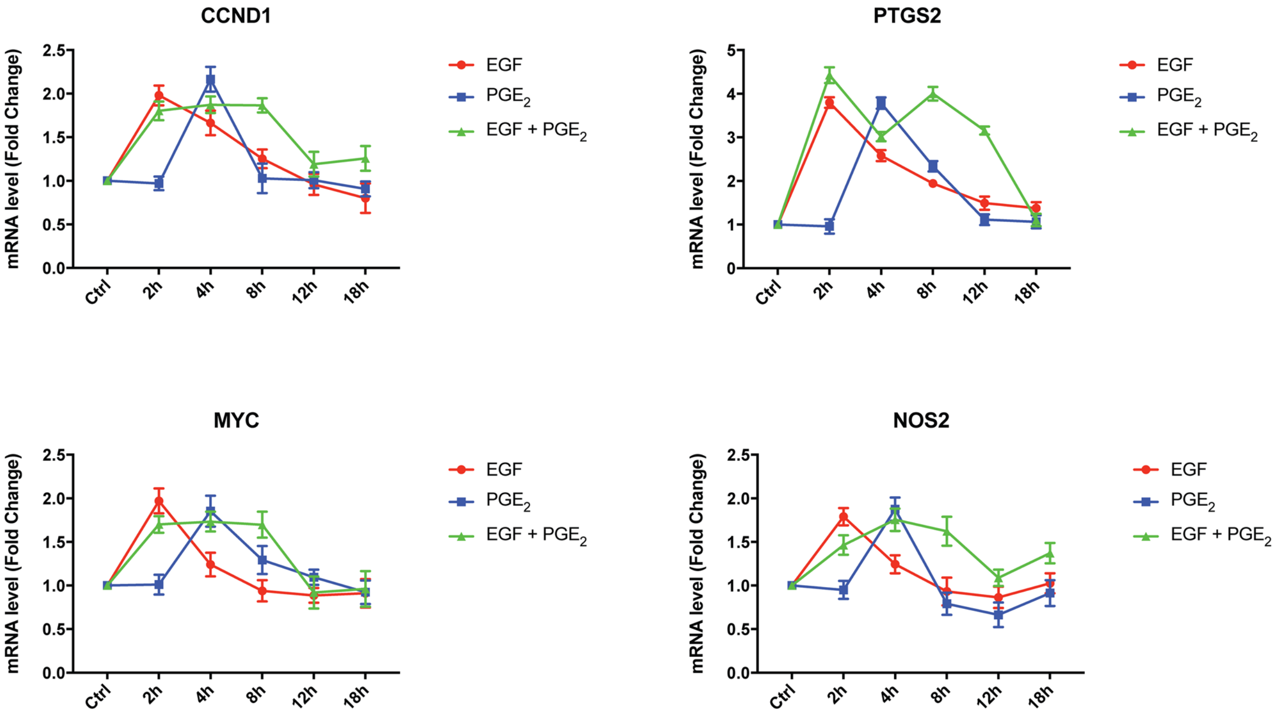

Supplementary Figure 6: EGF and PGE_2 induces the transcription of nuclear EGFR target genes in GLC82 up to 8 hours. GLC82 cells were starved overnight and then treated with 25ng/ml EGF or 1μM PGE_2 or the combination for 2, 4, 8, 12, 18 h. RNA was isolated and analyzed by qRT-PCR for a panel of nuclear EGFR target genes. The data are presented as mean of fold change ± SD of three independent experiments, relative to non-treated cells (Control), which were assigned to 1. Statistical analysis is reported in Supplementary Table 1.

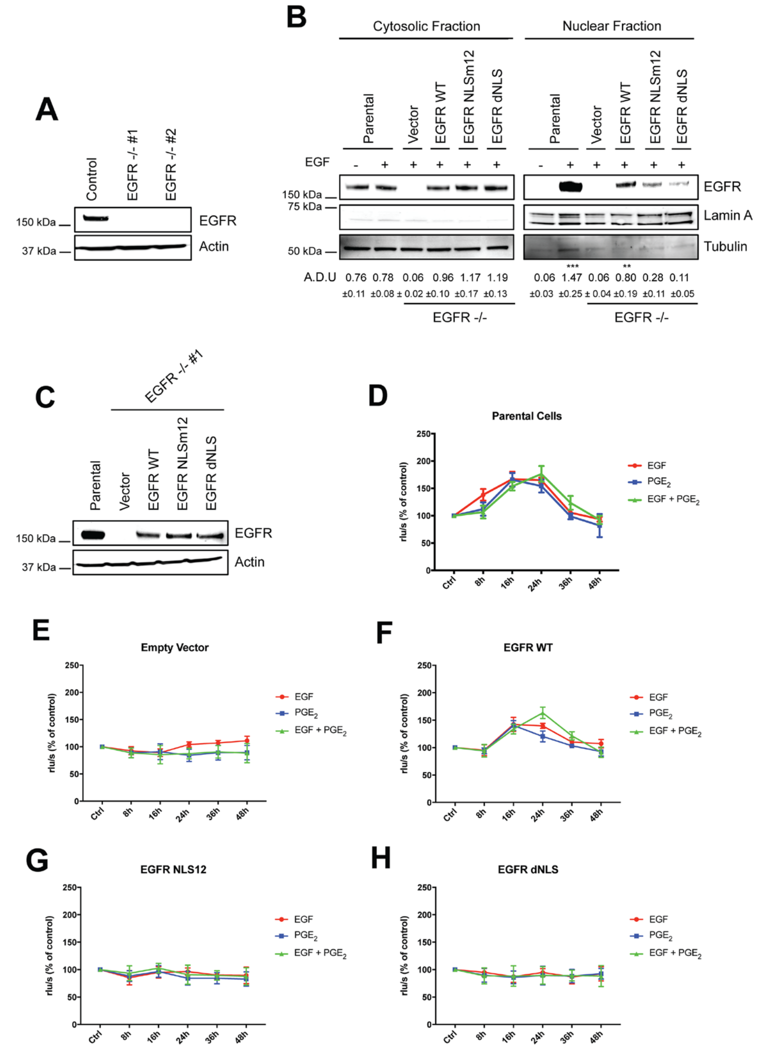

Supplementary Figure 7: EGF and PGE_2 induce nuclear EGFR-mediated GLC82 cell proliferation. **(A)** Immunoblotting analysis of EGFR expression in GLC82 wild type cells and two clones knockout for EGFR, Actin was used as loading control. **(B)** EGFR knockout GLC82 cells were transiently transfected with Vector or EGFR-WT or EGFR NLS mutant (NLSm12 or dNLS) plasmids for 48 h. Then EGFR nuclear import in response to 25ng/ml EGF for 10 min was analyzed by immunoblotting upon cell fractionation. Parental cells were included as a control. Tubulin and Lamin A were used as loading control for cytosolic and nuclear fraction respectively. Immunoblotting quantification was expressed in A.D.U. (arbitrary density unit) and as mean ± SD. $^{**}p < 0.01$, $^{***}p < 0.001$ vs Ctrl. EGFR in the cytoplasmic and nuclear fractions was normalized to Tubulin or Lamin A respectively. **(C)** Expression of EGFR in EGFR knockout cells transfected with Vector, EGFR-WT and NLS mutant plasmids for 96 h. **(D-H)** Parental GLC82 cells or EGFR -/- #1, #2 cells transfected with Vector or EGFR WT or EGFR NLS12 or EGFR dNLS mutant plasmids for 24 h were harvested and seeded for BrdU incorporation assay. Cell proliferation was assessed by measuring the luminescence after 8, 16, 24, 36, 48 h treatment with EGF or PGE_2 or the combination. Data are mean ± SD of triplicate cultures, expressed as % of control. Statistical analysis is reported in Supplementary Table 2.

Supplementary Table 1: Statistical analysis of nuclear EGFR target genes regulated by EGF and PGE_2 in GLC82 cells

CCND1					
	**2h**	**4h**	**8h**	**12h**	**18h**
EGF vs Ctrl	**0.0132 (*)**	**0.0424 (*)**	0.1422 (ns)	0.767 (ns)	0.3618 (ns)
PGE_2 vs Ctrl	0.7414 (ns)	**0.0144 (*)**	0.8837 (ns)	0.9466 (ns)	0.3896 (ns)
EGF+PGE_2 vs Ctrl	**0.0173 (*)**	**0.0116 (*)**	**0.0086 (**)**	0.3103 (ns)	0.2998 (ns)
EGF+PGE_2 vs EGF	0.3746 (ns)	0.346 (ns)	**0.0447 (*)**	0.339 (ns)	0.121 (ns)
EGF+PGE_2 vs PGE_2	**0.0244 (*)**	0.2273 (ns)	**0.0469 (*)**	0.3902 (ns)	0,0664 (ns)
PTGS2					
	**2h**	**4h**	**8h**	**12h**	**18h**
EGF vs Ctrl	**0.0019 (**)**	**0.0063 (**)**	**0.0097 (**)**	0.0852 (ns)	0.1077 (ns)
PGE_2 vs Ctrl	0.8266 (ns)	**0.0021 (**)**	**0.0076 (**)**	0.4551 (ns)	0.7069 (ns)
EGF+PGE_2 vs Ctrl	**0.0028 (**)**	**0.0025 (**)**	**0.0032 (**)**	**0.0018 (**)**	0.5326 (ns)
EGF+PGE_2 vs EGF	0.1017 (ns)	0.117 (ns)	**0.0077 (*)**	0.0114 (ns)	0.2983 (ns)
EGF+PGE_2 vs PGE_2	**0.0049 (**)**	**0.044 (*)**	**0.0135 (*)**	**0.0057 (**)**	0.8588 (ns)
MYC					
	**2h**	**4h**	**8h**	**12h**	**18h**
EGF vs Ctrl	**0.0209 (*)**	0.2186 (ns)	0.6673 (ns)	0.3154 (ns)	0.6399 (ns)
PGE_2 vs Ctrl	0.9348 (ns)	**0.0404 (*)**	0.2096 (ns)	0.3951 (ns)	0.626 (ns)
EGF+PGE_2 vs Ctrl	**0.0176 (*)**	**0.0234 (*)**	**0.0425 (*)**	0.7041 (ns)	0.8686 (ns)
EGF+PGE_2 vs EGF	0.2544 (ns)	0.1082 (ns)	0.0584 (ns)	0.8884 (ns)	0.8641 (ns)
EGF+PGE_2 vs PGE_2	**0.0427 (*)**	0.6278 (ns)	0.2048 (ns)	0.4812 (ns)	0.8906 (ns)
NOS2					
	**2h**	**4h**	**8h**	**12h**	**18h**
EGF vs Ctrl	**0.0154 (*)**	0.1439 (ns)	0.7117 (ns)	0.3801 (ns)	0.8395 (ns)
PGE_2 vs Ctrl	0.6713 (ns)	**0.0223 (*)**	0.2408 (ns)	0.141 (ns)	0.6198 (ns)
EGF+PGE_2 vs Ctrl	0.0528 (ns)	**0.0277 (*)**	0.0642 (ns)	0.4411 (ns)	0.0854 (ns)
EGF+PGE_2 vs EGF	0.1602 (ns)	0.0905 (ns)	0.0955 (ns)	0.2801 (ns)	0.1682 (ns)
EGF+PGE_2 vs PGE_2	0.0767 (ns)	0.5783 (ns)	0.0577 (ns)	0.2323 (ns)	0.797 (ns)

ns = non significant; * $p < 0.05$; ** $p < 0.01$.

Supplementary Table 2: Statistical analysis of BrdU incorporation assay in performed in GLC82 cells

	8h	16h	24h	36h	48h
**Parental Cells**					
	**8h**	**16h**	**24h**	**36h**	**48h**
EGF vs Ctrl	**0.0033 (**)**	**0.001 (***)**	**0.0008 (***)**	**0.0444 (*)**	0.0642 (ns)
PGE$_2$ vs Ctrl	0.1592 (ns)	**0.0009 (***)**	**0.0014 (**)**	0.7123 (ns)	0.2115 (ns)
EGF+PGE$_2$ vs Ctrl	0.3788 (ns)	**0.0003 (***)**	**0.0009 (***)**	**0.0316 (*)**	0.1505 (ns)
EGF+PGE$_2$ vs EGF	**0.0263 (*)**	0.2163 (ns)	0.2552 (ns)	0.076 (ns)	0.8121 (ns)
EGF+PGE$_2$ vs PGE$_2$	0.6068 (ns)	0.2396 (ns)	0.1087 (ns)	**0.0347 (*)**	0.4667 (ns)
**Empty Vector**					
	**8h**	**16h**	**24h**	**36h**	**48h**
EGF vs Ctrl	0.1616 (ns)	0.0574 (ns)	0.2028 (ns)	0.064 (ns)	0.0859 (ns)
PGE$_2$ vs Ctrl	0.1038 (ns)	0.3357 (ns)	0.0715 (ns)	0.2396 (ns)	0.2351 (ns)
EGF+PGE$_2$ vs Ctrl	0.0947 (ns)	0.217 (ns)	0.089 (ns)	0.2375 (ns)	0.3169 (ns)
EGF+PGE$_2$ vs EGF	0.5923 (ns)	0.7549 (ns)	0.0552 (ns)	0.0877(ns)	0.1148 (ns)
EGF+PGE$_2$ vs PGE$_2$	0.9716 (ns)	0.706 (ns)	0.7133 (ns)	0.8906 (ns)	0.9459 (ns)
**EGFR WT**					
	**8h**	**16h**	**24h**	**36h**	**48h**
EGF vs Ctrl	0.4603 (ns)	**0.0046 (**)**	**0.0008 (***)**	0.0811 (ns)	0.1751 (ns)
PGE$_2$ vs Ctrl	0.1211 (ns)	**0.0015 (**)**	**0.0225 (*)**	0.0881 (ns)	0.1965 (ns)
EGF+PGE$_2$ vs Ctrl	0.4308 (ns)	**0.0026 (**)**	**0.0005 (***)**	**0.0076 (**)**	0.189 (ns)
EGF+PGE$_2$ vs EGF	0.8996 (ns)	0.3924 (ns)	**0.0223 (*)**	0.1405 (ns)	0.0861 (ns)
EGF+PGE$_2$ vs PGE$_2$	0.9745 (ns)	0.4082 (ns)	**0.0064 (**)**	0.0159 (*)	0.873 (ns)
**EGFR NLS12**					
	**8h**	**16h**	**24h**	**36h**	**48h**
EGF vs Ctrl	0.1182 (ns)	0.4602 (ns)	0.3961 (ns)	0.0956 (ns)	0.2923 (ns)
PGE$_2$ vs Ctrl	0.1025 (ns)	0.6062 (ns)	0.0935 (ns)	0.0546 (ns)	0.0847 (ns)
EGF+PGE$_2$ vs Ctrl	0.439 (ns)	0.6234 (ns)	0.4079 (ns)	0.0969 (ns)	0.2469 (ns)
EGF+PGE$_2$ vs EGF	0.4978 (ns)	0.3884 (ns)	0.6226 (ns)	0.9439 (ns)	0.8864 (ns)
EGF+PGE$_2$ vs PGE$_2$	0.6185 (ns)	0.4847 (ns)	0.6294 (ns)	0.5296 (ns)	0.7032 (ns)
**EGFR dNLS**					
	**8h**	**16h**	**24h**	**36h**	**48h**
EGF vs Ctrl	0.2931 (ns)	0.0972 (ns)	0.2696 (ns)	0.1486 (ns)	0.3583 (ns)
PGE$_2$ vs Ctrl	0.2325 (ns)	0.105 (ns)	0.3329 (ns)	0.4004 (ns)	0.2551 (ns)
EGF+PGE$_2$ vs Ctrl	0.2478 (ns)	0.3345 (ns)	0.3172 (ns)	0.1272 (ns)	0.3460 (ns)
EGF+PGE$_2$ vs EGF	0.5336 (ns)	0.9361 (ns)	0.6233 (ns)	0.8117 (ns)	0.7746 (ns)
EGF+PGE$_2$ vs PGE$_2$	0.9205 (ns)	0.8691 (ns)	0.9898 (ns)	0.3848 (ns)	0.7442 (ns)

ns = non significant; * $p < 0.05$; ** $p < 0.01$; *** $p < 0.001$.

Supplementary Table 3: List of siRNA sequences

Target	Target Sequence (5′-3′)
AllStars Negative Control siRNA	-
Clathrin Heavy Chain 1A	AAGGAGAGTCTCAGCCAGTGA
Clathrin Heavy Chain 1B	TAATCCAATTCGAAGACCAAT
Caveolin 1A	AACTAAACACCTCAACGATGA
Caveolin 1B	AAGCATCAACTTGCAGAAAGA
Importin β1A	CTGGAATCGTCCAGGGATTAA
Importin β1B	AAGGGCGGAGATCGAAGACTA

Supplementary Table 4: List of qPCR primers

Name	Forward primer (5′-3′)	Reverse primer (5′-3′)
RPL19	GATGCCGGAAAAACACCTTG	TGGCTGTACCCTTCCGCTT
CCND1	GACCTTCGTTGCCCTCTGT	GGTTCAGGCCTTGCACTG
PTGS2	GCTTTATGCTGAAGCCCTATGA	TCCAACTCTGCAGACATTTCC
MYC	CACCAGCAGCGACTCTGA	CTGTGAGGAGGTTTGCTGTG
NOS2	GCTGCCAAGCTGAAATTGA	GATAGCGCTTCTGGCTCTTG
